# Humanized Murine Glioblastoma Models for Evaluation of Coxsackievirus Oncolytic Therapy

**DOI:** 10.3390/cancers18081280

**Published:** 2026-04-17

**Authors:** Yana D. Gumennaya, Marat P. Valikhov, Elizaveta R. Naberezhnaya, Pavel O. Vorobyev, Veronika V. Vadekhina, Olga N. Alekseeva, Anastasiia O. Sosnovtseva, Dmitry V. Kochetkov, Alesya V. Soboleva, Leen Ibrahim, Stepan A. Ionov, Gaukhar M. Yusubalieva, Alexander V. Ivanov, Peter M. Chumakov, Anastasia V. Poteryakhina

**Affiliations:** 1 Engelhard Institute of Molecular Biology, Russian Academy of Science, 119991 Moscow, Russia; enaberezhnaya2001@yandex.ru (E.R.N.); pvorobiev@eimb.ru (P.O.V.); olga_aleks@eimb.ru (O.N.A.); sosnovtseva@eimb.ru (A.O.S.); dkochet@eimb.ru (D.V.K.); asoboleva@eimb.ru (A.V.S.); leen.a98.ib@gmail.com (L.I.); stephan.ionov@yandex.ru (S.A.I.); aivanov@yandex.ru (A.V.I.); chumakov@eimb.ru (P.M.C.); alipatove@eimb.ru (A.V.P.); 2 Moscow Center for Advanced Studies, Kulakova Str. 20, 123592 Moscow, Russia; 3 Serbsky National Medical Research Center for Psychiatry and Narcology, Ministry of Health of the Russian Federation, 119034 Moscow, Russia; veronikavadehina@mail.ru; 4 Federal Scientific and Clinical Center for Specialized Types of Medical Care and Medical Technologies, Federal Medical and Biological Agency, 115682 Moscow, Russia; yusubalieva.gm@fccps.ru

**Keywords:** glioblastoma, syngeneic murine model, oncolytic virus, CXADR, enterovirus, LEV14, Coxsackievirus B5

## Abstract

**Background/Objectives**: Glioblastoma remains the most lethal primary brain tumor in adults, and progress in oncolytic virotherapy is limited by the lack of immunocompetent models permissive to human-tropic viruses. **Methods**: Here, murine CT-2A and GL261 glioma and B16 melanoma cell lines were engineered to express human Coxsackievirus and Adenovirus Receptor (CXADR) fused to tagBFP, generating “humanized” tumors that preserve parental growth characteristics while acquiring high susceptibility to group B Coxsackieviruses (CVBs) and adenovirus serotype 5. **Results**: CXADR expression in CT-2A, GL261, and B16 cells markedly enhanced binding, internalization, and replication of CVBs in vitro, with the strongest effect observed for LEV14 (attenuated CVB5), which reached up to 10^5^-fold higher viral titers in humanized cells compared with parental cells. Unchanged sensitivity to vesicular stomatitis virus indicated receptor-specific effects. Humanized CT-2A-CXADR-BFP and GL261-CXADR-BFP cells initiated aggressive subcutaneous and intracranial tumors in syngeneic C57BL/6 mice without signs of immune rejection, and histology and MRI confirmed invasive high-grade glioma phenotypes. In intracranial CT-2A-CXADR-BFP tumors, repeated intratumoral LEV14 administration induced extensive tumor necrosis and prolonged survival despite the rapid development of neutralizing antibodies. Systemic intravenous LEV14 dosing produced strong oncolytic activity against subcutaneous CT-2A-CXADR-BFP tumors, as demonstrated by pronounced tumor growth inhibition, long-lasting regression in a subset of animals with gliomas, and improved overall survival. **Conclusions**: Collectively, these data establish CXADR-humanized models as versatile, immunocompetent platforms for evaluation of CXADR-dependent oncolytic enteroviruses.

## Simple Summary

Glioblastoma is an aggressive brain cancer with very limited treatment options. Oncolytic viruses represent a promising therapy, but most available murine models cannot support infection by human-tropic viruses in an intact immune system. Here, we engineered widely used murine glioma cells to express a human virus entry receptor, creating “humanized” tumors that grow aggressively in immunocompetent mice while becoming highly sensitive to therapeutic enteroviruses. Using these models, we showed that a safe, non-pathogenic LEV14 Coxsackievirus strain can infect cancer cells, induce extensive tumor destruction in the brain following intratumoral injection, and significantly prolong survival even when antiviral immunity develops. These humanized models provide a practical platform for evaluating enterovirus-based therapies in realistic immune environments.

## 1. Introduction

Glioblastoma (GB) is the most treatment-resistant primary brain tumor in adults, with a median survival of 12–18 months and a 5-year survival rate of less than 10% [1,2,3]. GBs account for nearly 80% of primary malignant tumors in the central nervous system, posing significant challenges because of their invasive growth and high recurrence rates [4,5].

One promising therapeutic approach is the use of oncolytic viruses (OVs), which can selectively target and destroy cancer cells, including tumor-resident stem cells that drive GB recurrence [6,7]. Human GB cell lines are efficiently infected with non-pathogenic enterovirus strains, including the oncolytic Live Enterovirus Vaccine strains LEV14 (related to Coxsackievirus B5, CVB5) and LEV12 (related to Coxsackievirus B3, CVB3). Their anticancer activity has been extensively characterized in previous studies [8,9,10,11,12,13].

The development of new, effective OVs is often constrained by the lack of suitable preclinical models. Current murine syngeneic glioblastoma models, such as CT-2A and GL261, are not sensitive to human-tropic OVs because of species-specific differences in human enterovirus receptors that typically have orthologs rather than homologs in mice [14]. As a result, murine glioma cells are generally non-permissive to these viruses, and studies of oncolytic enteroviruses mostly rely on xenografts of human tumors in immunodeficient mice [15]. Although this strategy preserves viral-host compatibility, it does not reproduce both baseline and therapy-induced immune responses [16]. Currently, there are no syngeneic preclinical glioblastoma models suitable for testing human-specific oncolytic enteroviruses in immunocompetent mice.

To address this limitation, novel humanized murine glioblastoma models were developed and validated on the CT-2A and GL261 cell lines, which are among the most widely used models for evaluating anti-glioblastoma therapies in syngeneic mice [17,18,19,20,21]. In parallel, a humanized melanoma model based on the B16 melanoma cell line was generated to assess the oncolytic activity of human enteroviruses in a histologically distinct tumor type. These humanized tumor cell lines were engineered to express the human Coxsackievirus and Adenovirus Receptor (CXADR), which is essential for the entry of human group B Coxsackieviruses (CVBs) and multiple serotypes of human adenovirus [22,23].

In this paper, we report that mice with CXADR-expressing GBs or melanoma reproduce the sensitivity of human tumors to oncolytic enteroviruses, thereby providing a platform to evaluate their anticancer potential in an immunocompetent milieu.

## 2. Materials and Methods

### 2.1. Cell Cultures and Viral Strains

GL261 and CT-2A murine glioma cell lines were kindly provided by Dr. Aleksei A. Stepanenko (Department of Fundamental and Applied Neurobiology, Serbsky National Medical Research Center for Psychiatry and Narcology, Moscow, Russia). Murine melanoma B16, human glioma LN-18, LN-229, DBTRG-05MG, U251-MG cell lines, sublines of CT-2A, GL261, and B16, expressing tagBFP, HEK293T, and primary glioma cultures were obtained from the cell line collection of the Engelhardt Institute of Molecular Biology, Moscow, Russia. All cell lines, including newly generated CT-2A-CXADR-BFP, GL261-CXADR-BFP, and B16-CXADR-BFP, were cultured in DMEM (ServiceBio, Wuhan, China) supplemented with 10% FBS (HyClone, Cytiva, Logan, UT, USA), 2 mM glutamine, 100 U/mL penicillin, and 100 µg/mL streptomycin (PanEco, Moscow, Russia) at 37 °C in a humidified 5% CO_2_ atmosphere and split every 2–3 days.

The following non-pathogenic enterovirus vaccine strains were used: LEV14 strain of Coxsackievirus B5 (CVB5, GenBank: MG642820.1), Coxsackievirus B2 (CVB2, new uncharacterized strain), LEV12 strain of Coxsackievirus B3 (CVB3), Coxsackievirus B4 (CVB4, new uncharacterized strain), LEV15 strain of Coxsackievirus B6 (CVB6, GenBank: JQ041368.1), and Indiana strain of vesicular stomatitis virus (VSV-I). All enteroviruses and rhabdoviruses were obtained from the collection of the Engelhardt Institute of Molecular Biology, Moscow, Russia. Adenovirus strains Ad5-EGFP (Ad5-E1B55K-p2A-EGFP) and Ad5-RGD-EGFP (Ad5-pIIIa-EGFP (Fib-L5)) were obtained from the Department of Fundamental and Applied Neurobiology of Serbsky National Medical Research Center for Psychiatry and Narcology, Moscow, Russia. Both strains are replication-competent tumor-selective human adenovirus serotype 5 constructs with a 24-base pair deletion in the *E1A* gene and RGD motif-containing integrin-targeting peptide (RGD-4C) inserted into the fiber knob domain, enabling CXADR-independent cell entry [24]. EGFP fused through a p2A sequence to the C terminus of the E1B55K or pIIIa viral proteins downstream Fib-L5 region. All enterovirus strains were propagated in HEK293T-ΔIFNAR1, and adenoviruses were propagated in A549 cells. Viral titers (lgTCID50/mL) were determined by endpoint dilution using the Reed and Muench method [25].

### 2.2. Lentiviral Vector Construction and Transduction

The lentiviral vector pLCMV-CXADR-C-tagBFP-puro (Figure S1) was designed to express human CXADR fused with tagBFP. The construct harbored *CXADR* cDNA in-frame with a tagBFP reporter under the control of the CMV promoter, and a puromycin resistance gene controlled by the human *TP53* gene promoter (NP-promoter). Lentiviral stocks were produced as described previously [26]. Briefly, HEK293T-ΔIFNAR1 cells were co-transfected with packaging plasmids pLCMV-rev, pLCMV-gag-pol, and pLCMV-VSV-g together with pLCMV-CXADR-C-tagBFP-puro using GenJect-39 reagent (Molecta, Moscow, Russia). Virus-containing supernatants were collected every 12 h starting 24 h post-transfection. They were used to transduce CT-2A, GL261, and B16 cells. BFP-positive cells were selected with puromycin (2–5 μg/mL) through three sequential rounds (3 days each), and non-transduced CT-2A, GL261, and B16 cells were used as negative controls for selection.

### 2.3. Sensitivity to Oncolytic Viruses and Viral Reproduction

Cells were seeded in 96-well plates, and confluent monolayers were infected with 10-fold serial dilutions of a virus. Plates were incubated at 37 °C, 5% CO_2_, and 95% humidity. At 72 h post-infection, the cytopathic effect was evaluated by microscopy and quantified using a resazurin-based viability assay (alamarBlue™, Thermo Fisher Scientific, Waltham, MA, USA), and lgTCID50/mL values were calculated using the Reed and Muench method [25]. Viral replication kinetics were assessed by determining virus titers in supernatants collected 2, 6, 12, 24, 48, and 72 h post-infection at MOI 0.1 or 0.01. The titers (lgTCID50/mL) were measured on HEK293T-ΔIFNAR1 cells using 10-fold serial dilutions and the Reed and Muench method.

Live-cell imaging was performed using a JuLI™ Stage Real-Time Live Cell Imaging (NanoEntek, Seoul, Republic of Korea). Cells were seeded at equal confluence in 96-well plates and visualized to assess both viral cytopathic effects (48 or 72 h post-infection at MOI 10) and proliferation properties.

### 2.4. Subcutaneous and Intracranial Tumor Models and Therapy

Female 8-week-old immunocompetent C57BL/6 mice were implanted subcutaneously with 1.5 × 10^6^ CT-2A-CXADR-BFP or B16-CXADR-BFP cells in the dorsal region. Once tumors formed and reached an average volume of 50 mm^3^, mice were randomized into two groups (CT-2A-CXADR-BFP: *n* = 7, LEV14; *n* = 8, control; B16-CXADR-BFP: *n* = 5, LEV14; *n* = 5, control). In the CT-2A-CXADR-BFP model, 100 μL of purified LEV14 (10^6.6^ TCID50 per mouse) was administered intravenously via the tail vein at days 12, 15, 18, and 21 post-implantation; in the B16-CXADR-BFP model, 100 μL of purified LEV14 (10^7.4^ TCID50 per mouse) was administered intravenously at days 7, 10, 13, and 16. The control animals received PBS. Tumor growth was monitored by caliper linear measurements, and tumor volume was calculated as $\frac{1}{2}\times a^{2}\times b$, where a is the smaller orthogonal diameter [27]. The therapeutic efficacy index (T/C%) was defined as the ratio of the mean tumor volume in the treated group to that in the control group. Mice were euthanized once the tumor volume reached 2000 mm^3^.

For intracranial models of CT-2A, CT-2A-BFP, CT-2A-CXADR-BFP, or GL261-CXADR-BFP, cells were stereotactically implanted into the brains of female 8-week-old immunocompetent C57BL/6 mice under deep anesthesia induced by intraperitoneal injection of 20 mg/kg Zoletil and 5 mg/kg Xylazine, following published protocols [2,3,5,19,28,29,30,31,32,33,34,35,36,37]. For limiting dilution analysis, CT-2A-CXADR-BFP or GL261-CXADR-BFP cells (5 × 10^3^, 1.5 × 10^4^, 3 × 10^4^, 5 × 10^4^, or 1 × 10^5^) in 5 μL DMEM were stereotactically injected into the right or left hemisphere at coordinates relative to bregma: 2 mm lateral, 0.5 mm anterior, and 3.5 mm ventral. Cell suspensions in DMEM were loaded into a 100 μL Hamilton microsyringe with a 29G needle and injected at a rate of 1 μL/min. For therapy experiments, mice were implanted with 5 × 10^4^ CT-2A-CXADR-BFP, 5 × 10^4^ CT-2A-BFP, or 1.5 × 10^4^ CT-2A cells using the same procedure.

For the CT-2A-CXADR-BFP intracranial therapy cohort, treatment was initiated at day 10 post-implantation. At this time, mice were randomized into two groups (*n* = 10, LEV14 treatment; *n* = 10, control), and four intratumoral injections of 5 μL purified LEV14 (10^7.5^ TCID50 per mouse) were administered at days 10, 13, 16, and 19. The control mice received PBS. Tumor engraftment was confirmed by MRI (magnetic resonance imaging) at days 10 or 25 post-implantation. Animals were monitored and euthanized upon the development of neurological symptoms or a moribund condition. Brains from mice bearing CT-2A (*n* = 6), CT-2A-BFP (*n* = 3), or GL261-CXADR-BFP (*n* = 8) tumors were collected for histology to assess tumorigenesis efficiency.

In a separate experiment evaluating virus delivery, mice with CT-2A-CXADR-BFP tumors received a single intravenous or intratumoral injection of LEV14 (10^8^ TCID50 per mouse) at day 16 post-implantation. Mice were euthanized 30 min or 48 h after virus injection, and brains were harvested for immunohistochemical (IHC) analysis.

All animals used in this study were kindly provided by the vivarium of the Engelhardt Institute of Molecular Biology, Moscow, Russia. All procedures were approved by the Institutional Ethical Committee (Protocol No. 1 issued on 5 March 2025). Mice were housed under controlled temperature and humidity conditions with ad libitum access to food and water.

### 2.5. ICC and IHC Analyses

To assess viral internalization by immunocytochemistry (ICC), cells were seeded in 12-well plates and infected with LEV14 at MOI of 2. After 24 h, cells were washed with PBS, fixed in 2% paraformaldehyde at room temperature, permeabilized with 0.5% Triton X-100, and blocked with 2% BSA in PBS. For CXADR staining, the same protocol was used, except that cells were not infected and the permeabilization step was omitted. Cells were then incubated with either mouse monoclonal anti-hCXADR antibody (RHF63401, AntibodySystem, Paris, France; 1:300) or anti-LEV14 sheep antiserum (1:6000), followed by DyLight 488-conjugated goat anti-mouse IgG (405310, Biolegend, San Diego, CA, USA; 1:500) or FITC-conjugated mouse anti-goat/sheep IgG (F4891, Sigma Aldrich, Saint Louis, MO, USA; 1:1000), respectively. The LEV14 antiserum was obtained by immunizing six-month-old Dorper lambs from the Capri breeding farm (Kaluga region, Tarusa, Russia), as described previously [38]. Staining was performed according to the manufacturer’s instructions, and samples were analyzed using a Nikon A1R confocal laser-scanning microscope (Nikon, Tokyo, Japan).

To assess CXADR expression on the surface of parental and transduced glioma cell lines using flow cytometry, live cells were stained without permeabilization with primary anti-CXADR antibodies (RHF63401, AntibodySystem; 1:1000) and PE-conjugated rat anti-mouse IgG2a (407107, BioLegend; 1:1000). Cells were then harvested, and 30,000 events per sample were analyzed for fluorescence in the PE channel (561 nm excitation and 586/15 nm emission) on a BD LSRFortessa flow cytometer (BD Biosciences, Milpitas, CA, USA). Data were acquired using FACSDiva software version 8.0.1 (BD Biosciences) and analyzed with Flowing Software 2 (Turku Bioscience, Turku, Finland).

For histological analysis, subcutaneous and intracranial tumors from treated and control mice were collected at the end of the experiments, fixed in 4% paraformaldehyde at 4 °C overnight, and embedded in paraffin. Serial 5 μm sections were prepared using a rotary microtome (Leica Microsystems, Deerfield, IL, USA) and stained with hematoxylin and eosin (H&E) according to standard protocols [39].

To assess virus penetration in CT-2A-CXADR-BFP intracranial models by IHC, brain tissues were fixed overnight at 4 °C in 4% paraformaldehyde. Next, they were washed three times for 20 min in PBS and embedded in OCT compound, then snap-frozen at −80 °C. Coronal 10 μm cryosections were cut using a Microm HM 650 V cryostat (Microm International GmbH, Walldorf, Germany). Sections were permeabilized in 0.1% Triton X-100 for 15 min at room temperature, washed with PBS, and blocked with 1% goat serum for 1 h. Sections were incubated overnight at 4 °C with anti-LEV14 sheep antiserum (1:600), washed, and then incubated with FITC-conjugated mouse anti-goat/sheep IgG (F4891, Sigma Aldrich, 1:300) for 1 h at room temperature. Images were acquired using a Nikon A1R confocal laser scanning microscope (Nikon).

To analyze tumor immune infiltration in CT-2A-CXADR-BFP and B16-CXADR-BFP models, tumors were fixed overnight at 4 °C in 4% paraformaldehyde. Coronal 50 μm cryosections were cut using a Leica VT1200 S vibratome, permeabilized in 0.1% Triton X-100 for 30 min at room temperature, washed with PBS, and blocked with 1% goat serum for 1 h. Sections were incubated overnight at 4 °C with: Alexa Fluor 488 anti-mouse CD4 (100423, Biolegend), eFluor 660 CD8a (AMC908, eBioscience, San Diego, CA, USA), Brilliant Violet 421 anti-mouse NK1.1 (108731, Biolegend), and Brilliant Violet 421 anti-mouse Ly-6G (127627, Biolegend). Images were acquired using a Nikon A1R confocal laser scanning microscope (Nikon).

### 2.6. Magnetic Resonance Imaging

Magnetic resonance imaging (MRI) was used to confirm tumor engraftment and monitor growth. Mice were anesthetized with isoflurane, and T2-weighted images were acquired on a ClinScan 7T scanner (Bruker BioSpin, Ettlingen, Germany) using a field of view of 14 × 19 mm, slice thickness of 0.5 mm, and in-plane resolution of 384 × 288 pixels. T2-weighted coronal images with fat suppression were obtained with TR/TE = 370/46 mx, 3 averages, and echo-train length 18, with a total acquisition time of approximately 2 min. Tumors were visualized and manually contoured on consecutive slices in Vidar DICOM Viewer 3 software, and tumor volumes were calculated using the trapezoidal rule by averaging cross-sectional areas of adjacent slices and multiplying by slice thickness.

### 2.7. Virus Neutralization Microtests

Peripheral blood (50 μL) was collected from the tail vein of treated and control mice before therapy and 2 days after each virus injection. Blood samples were immediately mixed with EDTA and stored at −80 °C. Microneutralization assays were performed with minor modifications of published protocols [38,40,41]. Briefly, eight consecutive 2-fold serial dilutions of blood were prepared in 96-well round-bottom plates using serum-free DMEM and mixed with four consecutive 10-fold serial dilutions of virus, followed by incubation for 2 h at 37 °C, 5% CO_2_, and 95% humidity. The serum-virus mixtures were then transferred to pre-seeded HEK293T-ΔIFNAR1 monolayers. At 24 h post-infection, cytopathic effects were evaluated, and the neutralizing activity of each serum was defined as the minimum antiserum concentration at which more than 50% of cells remained viable at the selected MOI.

### 2.8. Peripheral Blood Analysis

Peripheral blood (500–1000 µL) was collected from treated and control mice bearing subcutaneous CT-2A-CXADR-BFP tumors at the indicated therapy timepoints and mixed with EDTA. Erythrocyte lysis was performed by incubation with 4 volumes of 0.8% NH_4_Cl in Milli-Q H_2_O. Leukocytes were resuspended in PBS + 0.5% BSA and blocked with FcR blocking reagent (Miltenyi Biotec, Bergisch Gladbach, Germany) for 15 min at 4 °C. Cells were stained using the following antibodies: Alexa Fluor 488 anti-mouse CD4 (100423, Biolegend), eFluor 660 anti-mouse CD8a (50-0081-82, Invitrogen, Carlsbad, CA, USA), PE/Cyanine7 anti-mouse/human CD11b (101216, Biolegend), and Brilliant Violet 421 anti-mouse Ly-6C (128032, Biolegend). Compensation was performed using single-stained controls. Samples were acquired on a BD LSRFortessa flow cytometer (BD Biosciences) and analyzed with Flowing Software 2 (Turku Bioscience). Lymphocytes were identified by FSC/SSC characteristics, and CD4+/CD8+ T cells were quantified as a percentage of lymphocytes. Monocytes were defined as CD11b+ cells with monocyte FSC/SSC characteristics and subdivided into classical (Ly6C high) and non-classical (Ly6C low) subsets [42].

### 2.9. RT-qPCR

Total RNA was isolated from cells grown on 6-well plates using ExtractRNA reagent and CleanRNA Standard kit (Evrogen, Moscow, Russia), followed by DNase I treatment (Thermo Fisher Scientific). cDNA synthesis was carried out using SuperScript III Reverse Transcriptase (Invitrogen) and random hexamer primers according to the manufacturer’s instructions. qPCR was performed on a CFX96 Touch System (Bio-Rad, Hercules, CA, USA) using qPCRmix-HS SYBR (Evrogen) and gene-specific primers (Table S1), which were validated for specificity by melting curve analysis (confirming single peaks without non-specific products) and for efficiency using serial cDNA dilutions, yielding R^2^ more than 0.98 for all primer pairs. The thermal cycling protocol was: 95 °C for 5 min; 40 cycles of 95 °C for 10 s, 66 °C for 15 s, and 72 °C for 15 s. Relative expression was calculated by the comparative ΔCt or ΔΔCt method [43], using murine *β-actin* or human ribosomal protein *L19* as internal controls. Fold changes in gene expression were calculated using the 2^−ΔΔCt^ method in parental and humanized murine cell lines.

### 2.10. Adenovirus Kinetics by Flow Cytometry

The replication efficiency of Ad5-EGFP and Ad5-RGD-EGFP was assessed by measuring EGFP-expressing cells. Cells were seeded in 12-well plates and infected with adenoviruses at MOIs of 0.01, 0.1, 1, or 10. At 48 h post-infection, cells were harvested, and 30,000 events per sample were analyzed for green fluorescence in the FITC channel (488 nm excitation and 530/30 nm emission) on a BD LSRFortessa flow cytometer (BD Biosciences). Data were acquired using FACSDiva software version 8.0.1 (BD Biosciences) and analyzed with Flowing Software 2 (Turku Bioscience).

### 2.11. Assessment of Released Cytokines

Evaluation of cytokines in serum of treated and control mice prior to therapy and 2 days following each virus injection was performed using a multiplex bead-based system (LEGENDplex™): Mouse Th Cytokine panel (12-plex, cat# 740143, Biolegend) and Mouse Cytokine Panel 2 (13-plex, cat# 740134, Biolegend). 25 cytokines were assessed: IL-2, 4, 5, 6, 9, 10, 13, 17A, 17F, 22, IFN-γ, TNF-α, IL-1α, IL-1β, IL-3, IL-7, IL-11, IL-12p40, IL-12p70, IL-23, IL-27, IL-33, IFN-β, GM-CSF, and TSLP. All experiments were performed following the manufacturer’s instructions, and data were analyzed using LEGENDplex™ Data Analysis Software (version 2025-05-01).

### 2.12. Statistical Analysis

Data are presented as mean ± SD. All experiments were performed in at least three independent biological replicates. Statistical analyses were carried out using the Mann–Whitney U test for group comparison and the log-rank test for survival analysis. Differences were considered statistically significant at: *, *p* < 0.05; **, *p* < 0.01; ***, *p* < 0.001. Graphs and statistical analyses were generated using GraphPad Prism version 8.0.2 (GraphPad Software).

## 3. Results

### 3.1. Generation of Humanized CT-2A, GL261, and B16 Lines Expressing CXADR

**Figure 1:**
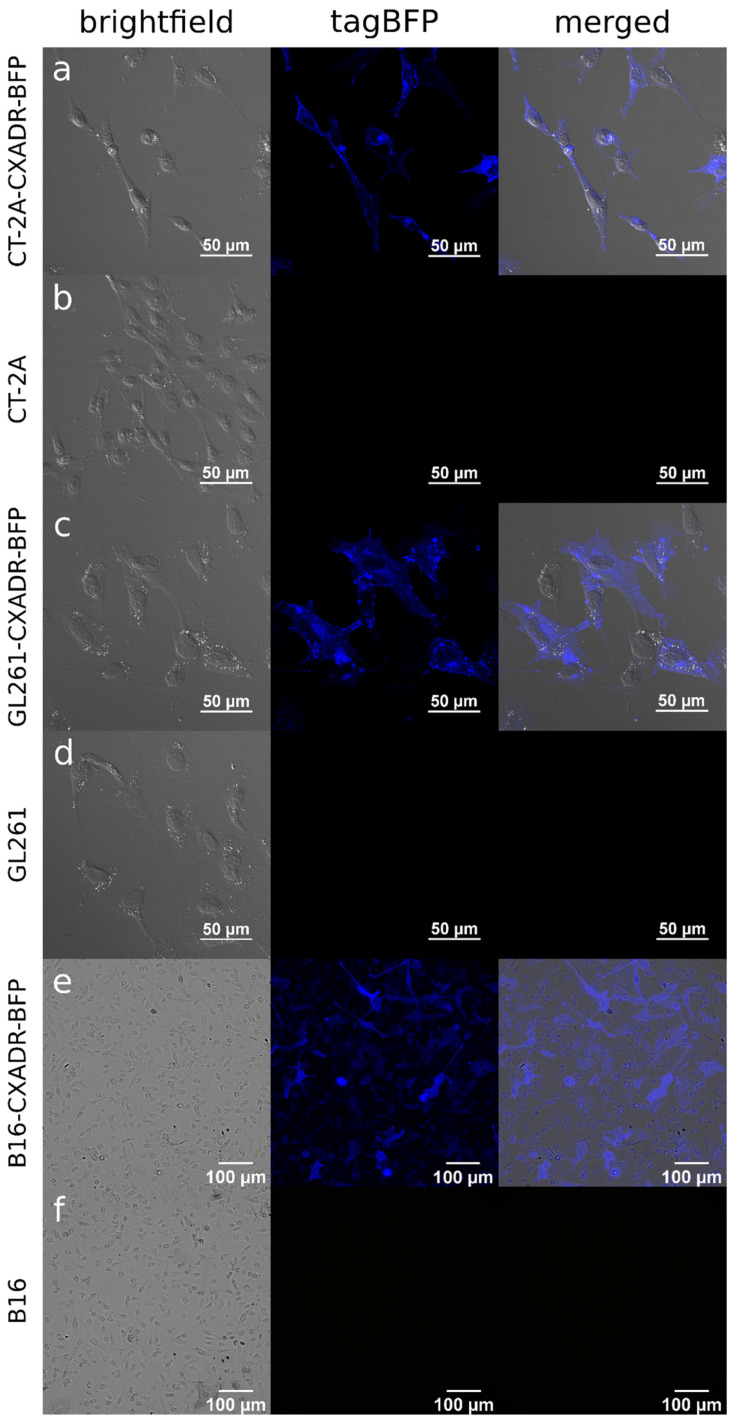
TagBFP fluorescence in humanized murine cell lines. (**a**) CT-2A-CXADR-BFP (×40); (**b**) CT-2A (×40); (**c**) GL261-CXADR-BFP (×40); (**d**) GL261 (×40); (**e**) B16-CXADR-BFP (×20); (**f**) B16 (×20). Brightfield, tagBFP, and merged channels are shown in the panels.

Murine CT-2A, GL261, and B16 cells stably expressing human CXADR were generated by lentiviral transduction followed by three rounds of puromycin selection. The morphology and proliferation rate of the transduced lines (CT-2A-CXADR-BFP, GL261-CXADR-BFP, and B16-CXADR-BFP) remained consistent with their parental lines (Figure 1, Figures S2 and S3). The lentiviral construct (Figure S1) encoded the human *CXADR* gene fused in-frame to the blue fluorescent protein (tagBFP). Blue fluorescence signal thus allowed visualization of CXADR expression and localization, which were further confirmed by ICC analysis. Parental CT-2A, GL261, and B16 cells showed neither staining for human CXADR nor detectable tagBFP fluorescence, whereas humanized cells exhibited robust blue fluorescence at both the plasma membrane and intracellular compartments and positive CXADR immunostaining (Figure 1 and Figure S2), confirming efficient transduction and stable expression of human CXADR in murine cells.

RT-qPCR analysis further confirmed *CXADR* mRNA expression in CT-2A-CXADR-BFP, GL261-CXADR-BFP, and B16-CXADR-BFP lines. Expression levels were calculated as log_2_(fold change) using the ΔΔCt method, with murine *β-actin* as a housekeeping control (Figure S4a and Table S1). Amplification of the transgene and Sanger sequencing of the product confirmed the integrity of the human *CXADR* coding sequence in all transduced lines (Table S2). ICC analysis performed by flow cytometry confirmed hyperexpression of human CXADR on the surface of glioma cells (Figure S4c–e). *CXADR* expression was also evaluated in model and primary human glioma cell lines susceptible to Coxsackieviruses, using the ΔCt method with *RPL-19* as a housekeeping gene (see Figure S4b and Table S1). Notably, CXADR expression in human glioblastoma cells was heterogeneous, reflecting the variability observed among the murine humanized lines.

### 3.2. Enhanced Susceptibility of Humanized Lines to Coxsackievirus and Adenovirus Infection

**Figure 2:**
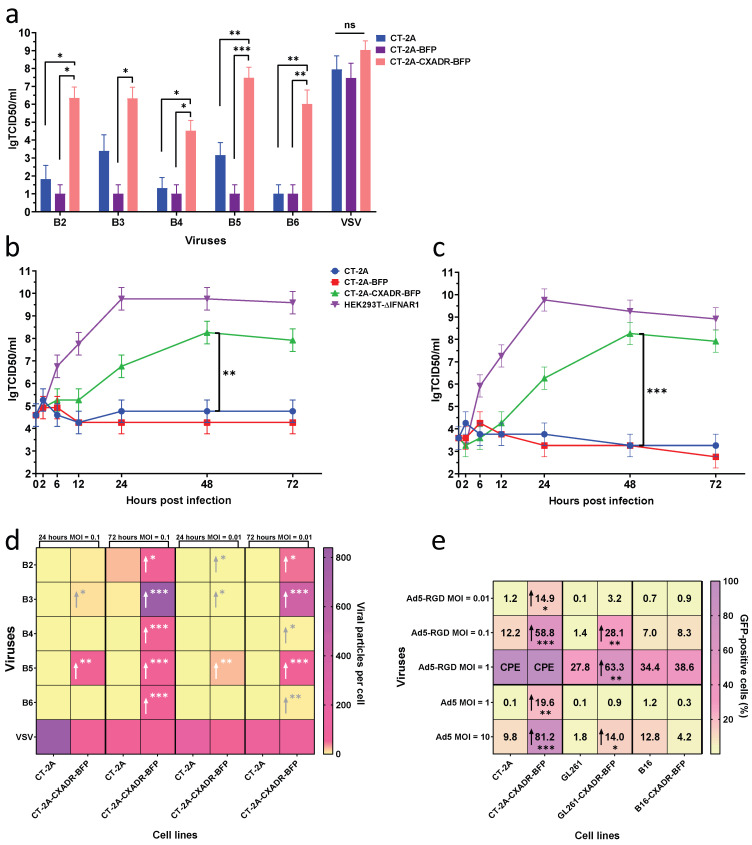
Increased susceptibility of humanized cell lines to Coxsackievirus and adenovirus infection. (**a**–**d**) Viral replication efficiency of CVBs and VSV in CT-2A, CT-2A-BFP, and CT-2A-CXADR-BFP cell lines. (**a**) Sensitivity of cells to viruses was evaluated at 72 h after infection. CVB5 replication was measured 2, 6, 12, 24, 48, and 72 h after infection at MOI 0.1 (**b**) or MOI 0.01 (**c**). (**d**) Viral replication of CVBs and VSV was measured at 24 h and 72 h after infection. (**e**) Adenovirus replication (Ad5-EGFP and Ad5-RGD-EGFP) was analyzed by flow cytometry at 48 h post-infection at different MOIs in CT-2A, CT-2A-CXADR-BFP, GL261, GL261-CXADR-BFP, B16, and B16-CXADR-BFP cell lines. The cytopathic effect (CPE), indicative of complete cell death due to adenoviral infection, is shown. Arrows in (**d**,**e**) denote statistically significant increases relative to the corresponding parental cell line. The data are shown as means ± SD of three independently related experiments (*n* = 3). Statistical analysis was performed using the Mann–Whitney U-test, * *p* < 0.05, ** *p* < 0.01, *** *p* < 0.001.

To assess if humanized cell models are permissive to oncolytic viruses, sensitivity and production of infectious progeny were quantified by determining lgTCID50/mL values. The CT-2A-CXADR-BFP line exhibited markedly increased sensitivity to the LEV14 strain (CVB5), and other CVB serotypes 72 h post-infection compared with parental CT-2A cells (Figure 2a). In contrast, CXADR expression did not significantly affect susceptibility to vesicular stomatitis virus (VSV), which does not utilize CXADR as an entry receptor (Figure 2a). These data indicate that CXADR expression specifically enhances enterovirus entry without exerting negative non-specific effects on cell viability. Cytopathic effects (CPE) in parental and humanized cell lines 48 h or 72 h after infection with CVBs or VSV at MOI 10 and MOI 1, respectively, are shown in Supplementary Videos S1 and S2. Humanized CT-2A-CXADR-BFP and GL261-CXADR-BFP lines exhibited extensive cytopathic destruction by the end of the observation period (Videos S1e,f and S2d), whereas parental lines remained resistant to CVB infection (Videos S1a,b and S2a). VSV infectivity was similar in CXADR-positive and CXADR-negative counterparts (Videos S1c,g and S2b,e).

CXADR expression also increased CVB, but not VSV, replication (Figure 2d). In CT-2A-CXADR-BFP cells, total CVB5 yield after infection at MOI 0.1 peaked at 48 h, reaching approximately 10^3.7^-fold and 10^4^-fold higher titers than in CT-2A and CT-2A-BFP cells, respectively. At MOI 0.01, peak viral yield at 48 h was about 10^5^-fold higher in CT-2A-CXADR-BFP cells than in CT-2A and CT-2A-BFP cells (Figure 2b,c). Comparable increases in infectivity and viral replication were observed in GL261-CXADR-BFP and B16-CXADR-BFP lines (Figure S5), underscoring the critical role of human CXADR expression on the cell surface in supporting efficient enterovirus infection.

To further validate the humanized models, susceptibility to adenovirus infection was examined, given that CXADR functions as a primary receptor for both CVBs and several adenovirus serotypes, including serotype 5. Infection kinetics of Ad5-EGFP and Ad5-RGD-EGFP were analyzed by flow cytometry based on green fluorescence detectable in infected cells. CT-2A-CXADR-BFP and GL261-CXADR-BFP lines displayed a significantly higher proportion of EGFP-positive cells at all tested MOIs than their parental counterparts, confirming increased susceptibility to Ad5 infection. In contrast, B16 and B16-CXADR-BFP lines showed no significant difference in the fraction of infected cells, suggesting the presence of additional cell-intrinsic restriction factors that limit adenoviral replication despite expression of the receptor at the cell surface (Figure 2e). This observation is consistent with prior data indicating that expression levels of adenoviral receptors alone do not always confer adenovirus-mediated cytolysis [44].

**Figure 3:**
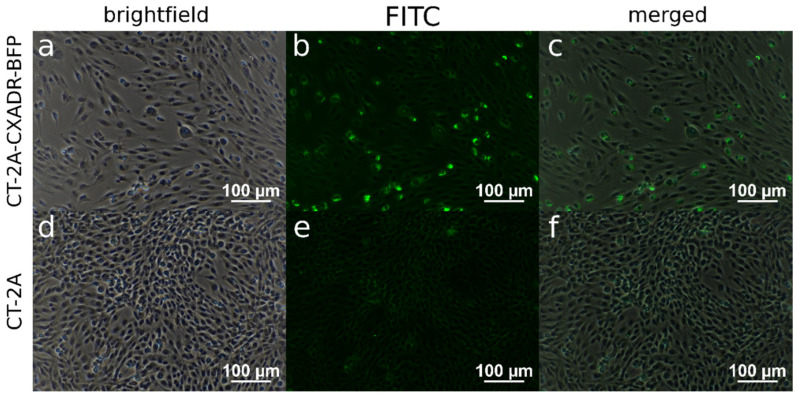
LEV14 internalization in glioma cells. ICC staining of fixed CT-2A-CXADR-BFP (**a**–**c**) and CT-2A (**d**–**f**) cells at 24 h after infection with LEV14 at MOI 2 (×20). Sheep antiserum against LEV14 was used as the primary antibody, and FITC-labeled anti-sheep IgG (Fc-specific) served as the secondary antibody. Brightfield, FITC, and merged channels are shown in the panels.

To visualize CXADR-dependent LEV14 internalization, ICC staining was performed using a sheep anti-LEV14 antiserum (Figure 3). The antibodies stained virions on the cell surface and inside the cells, confirming LEV14 binding and internalization in the CT-2A-CXADR-BFP cells (Figure 3a–c). In contrast, no LEV14 internalization was detected in the parental CT-2A line (Figure 3d–f).

### 3.3. Evaluation and Characterization of Humanized Tumor Models

**Figure 4:**
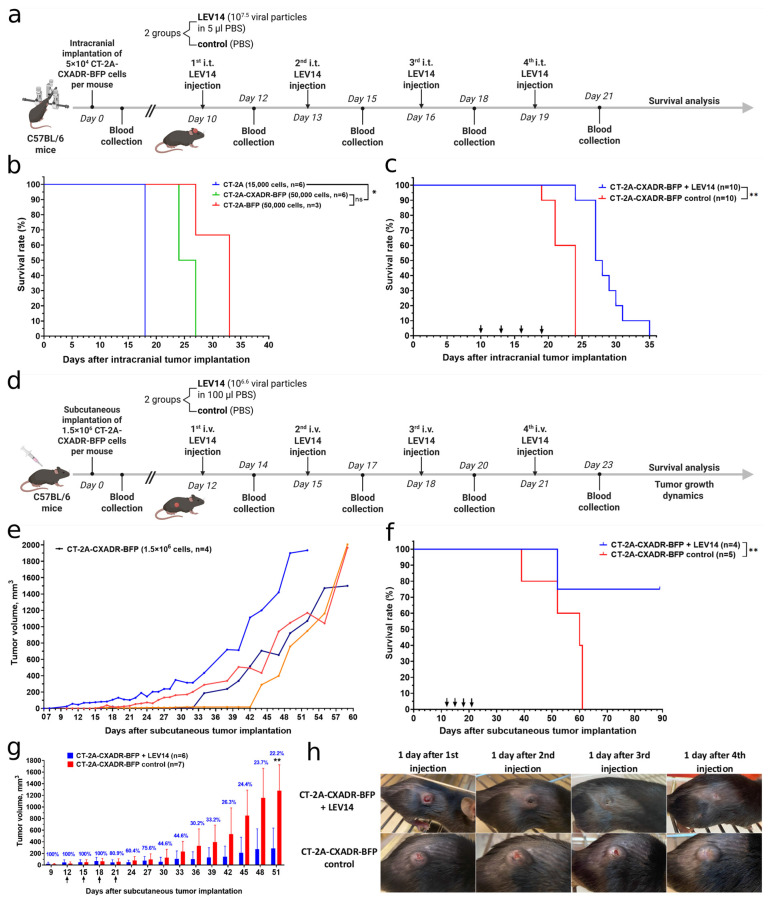
Oncolytic activity of LEV14 in orthotopic and subcutaneous humanized glioma models. (**a**) Experimental scheme for intracranial tumor implantation followed by intravenous LEV14 treatment (created with Biorender.com). (**b**) Kaplan–Meier survival curves of mice bearing intracranial CT-2A, CT-2A-BFP, and CT-2A-CXADR-BFP tumors. (**c**) Kaplan–Meier survival curves of mice bearing intracranial CT-2A-CXADR-BFP tumors treated with four consecutive intratumoral administrations of LEV14 (at days 10, 13, 16, and 19 post tumor implantation, 10^7.5^ TCID50 per mouse). (**d**) Experimental scheme for subcutaneous tumor implantation followed by intratumoral LEV14 treatment (created with Biorender.com). (**e**) Growth dynamics of individual subcutaneous CT-2A-CXADR-BFP tumors. Each colored curve corresponds to a distinct biological replicate within the group. (**f**) Kaplan–Meier survival curves of mice bearing subcutaneous CT-2A-CXADR-BFP tumors treated with four consecutive intravenous administrations of LEV14 (at days 12, 15, 18, and 21 post tumor implantation, 10^6.6^ TCID50 per mouse). (**g**) Growth dynamics of CT-2A-CXADR-BFP subcutaneous xenografts following four consecutive intravenous administrations of LEV14 (at days 12, 15, 18, and 21 post-implantation, 10^6.6^ TCID50 per mouse). T/C—tumor/control efficacy index, %. Arrows in (**c**,**f**,**g**) indicate the days of virus administration. Values are means ± SD. (**h**) Comparison of tumor growth between LEV14-treated and control CT-2A-CXADR-BFP subcutaneous xenografts. n, number of animals. Statistical analysis was performed using the Mann–Whitney U-test or the log-rank test, * *p* < 0.05, ** *p* < 0.01.

Next, tumorigenicity and engraftment of subcutaneous and intracranial glioblastoma and melanoma models were evaluated in immunocompetent C57BL/6 mice. Optimal cell numbers for reliable tumor formation were determined by limiting dilution analysis (Figure S6). Subcutaneous implantation of 1.5 × 10^6^ CT-2A-CXADR-BFP cells into the dorsal region consistently led to tumor formation, with a mean volume of 1156 ± 441 mm^3^ at 7 weeks post-implantation (Figure 4e). Histological examination of the resulting tumors (Figures S7 and S8) revealed malignant, invasive growth patterns in both CT-2A-CXADR-BFP and B16-CXADR-BFP subcutaneous models. CT-2A-CXADR-BFP tumors showed early invasion into the surrounding connective tissue (Figure S7a) and increased cell density (Figure S7a,b), which is typical for this type of cancer. At later time points, these tumors displayed further increases in cell density (Figure S7c), histological heterogeneity (Figure S7d), and notable invasion into adjacent normal tissues (Figure S7e,f). Similarly, B16-CXADR-BFP tumors consisted of densely packed atypical cells with clear infiltration into the surrounding stroma and adipose tissue, consistent with an invasive melanoma phenotype (Figure S8).

In intracranial glioma models, stereotactic injection into the striatum of 5 × 10^4^ CT-2A-CXADR-BFP or CT-2A-BFP cells, or 1.5 × 10^4^ CT-2A cells, in all cases resulted in tumor formation, conferring 100% mortality (Figure 4b). Histology and MRI confirmed progressive tumor growth with characteristic features of high-grade glioma (Figures S9 and S10). The kinetics of tumor growth differed between CT-2A, CT-2A-BFP, and CT-2A-CXADR-BFP tumors: transgene-expressing cells induced more slowly growing tumors than parental CT-2A cells. By day 10 after implantation, CT-2A tumors were already clearly visible (Figure S10a–c), whereas CT-2A-BFP tumors remained undetectable, and CT-2A-CXADR-BFP tumors were detectable but substantially smaller (Figure S10d,e). The delayed growth of CT-2A-BFP tumors likely reflects increased immunogenicity. Kaplan–Meier analysis showed median survival times of 18-, 33-, and 25.5-days post-implantation for mice bearing CT-2A, CT-2A-BFP, and CT-2A-CXADR-BFP tumors, respectively (Figure 4b). Likewise, intracranial injection of GL261-CXADR-BFP cells resulted in consistent tumor formation and eventual death (Figure S6b). Histological examination demonstrated sustained tumor infiltration and progression (Figure S11). Overall, despite expression of human CXADR or BFP, the modified glioma cell lines reliably established aggressive intracranial tumors in syngeneic C57BL/6 mice, with no evidence of immune rejection or spontaneous regression.

### 3.4. LEV14 Therapy Prolongs Survival

To assess LEV14 efficacy in a humanized in vivo setting, immunocompetent C57BL/6 mice bearing intracranial or subcutaneous CT-2A-CXADR-BFP tumors, or subcutaneous B16-CXADR-BFP tumors, were treated with the LEV14 (CVB5) strain.

**Figure 5:**
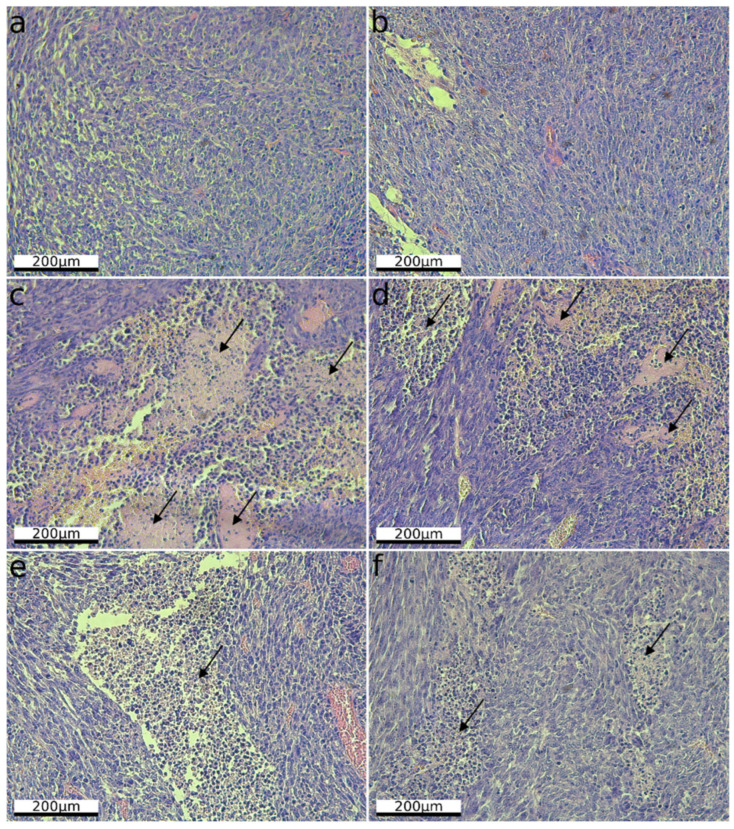
Histological examination (H&E staining) of CT-2A-CXADR-BFP orthotopic glioma after intratumoral LEV14 therapy. (**a**,**b**) Brain sections from control mice without therapy (×20). (**c**–**f**) Brain sections from mice treated with LEV14 (×20). Arrows indicate extensive areas of necrosis and regions with loss of cellular architecture.

For the orthotopic glioma model, four intratumoral injections of LEV14 (5 μL of purified virus containing 10^7.5^ TCID50 per mouse) were administered at 3-day intervals to evaluate oncolytic activity (Figure 4a). Neutralizing activity of sera from LEV14-treated mice showed high neutralizing capacity developing as early as the second virus administration (Figure S12a). Nevertheless, substantial oncolytic effects were observed. LEV14-treated mice exhibited significantly prolonged survival compared with controls (Figure 4c). Histological examination (Figure 5) revealed densely packed viable CT-2A-CXADR-BFP cells in control tumors (Figure 5a,b), whereas LEV14-treated tumors showed extensive necrosis (Figure 5c,d), widespread cellular destruction, and a reduction in viable cell density (Figure 5c–f). IHC analysis further revealed enhanced immune cell infiltration in LEV14-treated CT-2A-CXADR-BFP tumors, with 2.5-fold increased CD8+ T cells, and 6.4-fold increased Ly6G+ neutrophils 4 days post-final dose compared to controls (Figure S13), confirming the induction of a proinflammatory tumor microenvironment.

Mice with subcutaneous CT-2A-CXADR-BFP tumors received four intravenous injections of LEV14 (10^6.6^ TCID50 in 100 μL of PBS per dose) at 3-day intervals to evaluate systemic antitumor effects (Figure 4d). LEV14 treatment significantly inhibited tumor growth (Figure 4g). Therapy resulted in a strong oncolytic effect against CT-2A-CXADR-BFP cells, yielding a final T/C value of 22.2% and approximately a 4.5-fold reduction in tumor growth rate. Survival analysis showed marked benefit in the LEV14-treated group, which exhibited no deaths up to day 52 post-implantation and an overall survival rate of 75% (Figure 4f). Remarkably, complete tumor regression was observed in a subset of animals as early as 1 day after the last virus dose and persisted without relapse throughout the 90-day follow-up (Figure 4h). As with intratumoral dosing, neutralizing antibodies developed in response to repeated intravenous LEV14 injections (Figure S12b), yet sustained oncolysis was maintained over the observation period. Histological evaluation (Figure S14) of tumors from LEV14-treated mice revealed extensive confluent necrosis with acellular areas (Figure S14c,d,f) and, in some instances, complete regression of tumor tissue (Figure S14e). Peripheral blood analysis revealed modest CD8+ T cell elevation and monocyte increase during LEV14 therapy (Figure S15).

Mice with subcutaneous B16-CXADR-BFP melanoma received four intravenous LEV14 injections (10^7.4^ TCID50 in 100 μL of PBS per dose) at 3-day intervals (Figure S16a). Treatment led to inhibition of tumor growth (Figure S16b). Histological analysis (Figure S17) of melanoma tissues from LEV14-treated animals demonstrated extensive necrotic regions and pronounced tumor cell damage (Figure S17c–f). IHC analysis confirmed significant immune activation, revealing 2.2-fold higher CD8+ T cell infiltration, 3.6-fold higher Ly6G+ neutrophil infiltration, and 2.1-fold higher CD4+ T cell infiltration 4 days after the final dose compared to controls (Figure S18), demonstrating effective remodeling of the proinflammatory microenvironment.

Serum cytokine profiling further characterized LEV14-induced immune activation across CT-2A-CXADR-BFP tumor models (Figure S19). In subcutaneous tumors, significant TNF-α elevation occurred specifically after the final dose alongside IL-6 peaks following the second and third administrations, while IFN-γ increased early transiently after the first dose. Conversely, intracranial tumors showed no detectable TNF-α or IL-6—consistent with CNS immune privilege—but exhibited robust IL-2 surges, prominent IL-9 elevation post-third/fourth doses, and early IL-12p40 increases. These data demonstrate compartmentalized proinflammatory activation with peripheral innate responses (TNF-α, IL-6) dominating subcutaneous tumors and sustained CNS T cell engagement (IL-2, IL-9) driving orthotopic efficacy.

### 3.5. Viral Penetration into Experimental Glioma

**Figure 6:**
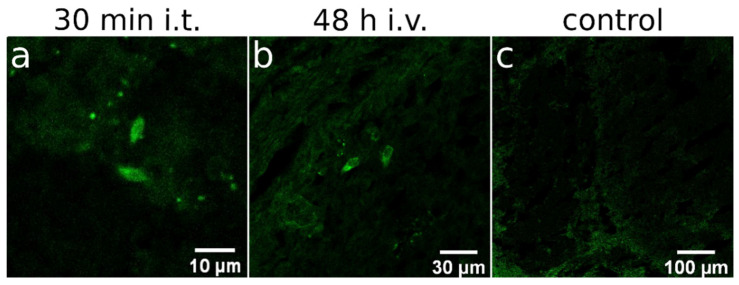
IHC detection of LEV14 in CT-2A-CXADR-BFP gliomas. CT-2A-CXADR-BFP tumor-bearing mice received a single dose of LEV14 either intratumorally (**a**) or intravenously (**b**). Brain sections collected at 30 min (**a**) or 48 h (**b**) post-infection were stained with sheep anti-LEV14 primary antiserum and FITC-conjugated anti-sheep secondary antibody (×20). (**c**) Control section processed without primary antibody. FITC signal is shown in the panels.

To determine whether LEV14 can reach intracranial tumors following local or systemic administration, IHC analysis was performed on brain sections from CT-2A-CXADR-BFP glioma-bearing mice. At day 16 after tumor implantation, animals received a single dose of LEV14 (10^8^ TCID50 per mouse) administered either intratumorally or intravenously, and brains were collected at predefined time points for staining with sheep anti-LEV14 antiserum (Figure 6). After intratumoral injection, viral capsid signal was detected within the tumor mass as early as 30 min post-administration (Figure 6a), indicating rapid local distribution. Following intravenous injection, LEV14 became detectable within tumors at 48 h post-administration (Figure 6b). In this setting, viral antigen was primarily observed in perivascular regions and at the tumor periphery (Figure 6b), consistent with delivery from the circulation into the lesion. These observations show that LEV14 can reach orthotopic gliomas after intravenous administration and support further evaluation of intravenous dosing as a complement to direct intratumoral delivery. The appearance of LEV14 within tumors at 48 h after dosing defines a practical time point for future studies exploring systemic therapeutic regimens.

## 4. Discussion

Here, we established CXADR-expressing humanized syngeneic glioma and melanoma models to evaluate how the Coxsackievirus and Adenovirus Receptor (CXADR) confers susceptibility to oncolytic enteroviruses. We further demonstrated that the CT-2A-CXADR-BFP model is suitable for preclinical evaluation of oncolytic virotherapy using CXADR-dependent enteroviruses such as LEV14.

Immunocytochemistry and tagBFP fluorescence revealed heterogeneous subcellular distribution of human CXADR in the engineered cell lines, with staining detectable not only at the plasma membrane but also in intracellular compartments (see Figure 1 and Figure S2). This subcellular localization pattern was most pronounced in GL261-CXADR-BFP cells, where the tagBFP signal was predominantly intracellular (Figure 1c), and CXADR immunostaining (performed even without permeabilization) primarily labeled cytoplasmic vesicles and internal compartments (Figure S2c). This localization may reflect constitutive endocytosis of overexpressed membrane receptors [45], a process that sequesters functional receptors away from the cell surface into intracellular vesicles. Consistent with this interpretation, GL261-CXADR-BFP cells expressed substantially higher CXADR mRNA and protein levels than CT-2A-CXADR-BFP and B16-CXADR-BFP cells (Figure S4), in line with greater intracellular accumulation of the CXADR-BFP fusion protein. By contrast, CT-2A-CXADR-BFP and B16-CXADR-BFP showed less intracellular signal and more membrane-associated staining, consistent with their lower expression levels.

Interestingly, total CXADR expression levels did not correlate directly with LEV14 susceptibility across the models. Although GL261-CXADR-BFP displayed the highest expression, it showed the smallest increase in viral sensitivity, whereas B16-CXADR-BFP (with the lowest CXADR expression) demonstrated the most pronounced enhancement (Figure 2a and Figure S5a,b). Several mechanisms may explain this discrepancy. First, differential activation of antiviral interferon pathways in GL261 cells may limit viral replication despite efficient entry [46]. Second, prior studies have shown that even minimal CXADR expression can be sufficient for effective infection, suggesting a saturation threshold beyond which higher expression yields no additional viral entry [10]. Third, surface CXADR availability, rather than total expression level, may be the key determinant. As noted above, B16-CXADR-BFP and CT-2A-CXADR-BFP cells with lower overall CXADR expression retain a higher fraction of receptor at the plasma membrane, thereby enhancing viral access. Our data therefore suggest that relatively low CXADR expression may be sufficient to support efficient LEV14 entry once a minimal surface density threshold is reached, whereas further overexpression may preferentially increase intracellular sequestration rather than productive surface binding sites.

In vivo, the subcutaneous CT-2A-CXADR-BFP model exhibited a clear therapeutic response to LEV14 (Figure 4c,f–h). In the B16-CXADR-BFP subcutaneous melanoma model, LEV14 showed more modest antitumor activity (Figure S16b), implying that factors beyond receptor presence modulate viral sensitivity. This finding parallels the comparable adenoviral susceptibility observed between B16 and B16-CXADR-BFP cells (Figure 2e), underscoring that CXADR expression alone does not determine the outcome of coxsackievirus or adenovirus infection, either in vitro or in vivo. Together, these observations highlight a complex interplay of cell-intrinsic determinants such as antiviral signaling, receptor distribution, and intracellular permissiveness in shaping oncolytic efficacy.

To ensure that CXADR expression did not compromise in vivo tumorigenicity, we compared growth kinetics between humanized and parental cell lines. All transgenic lines formed tumors with 100% efficiency, without spontaneous regression, indicating that the transgenes did not induce immunorejection. The CT-2A-CXADR-BFP model, our principal in vivo platform, exhibited survival dynamics consistent with literature reports for parental CT-2A tumors at comparable inoculum doses. For instance, intracranial implantation of 5 × 10^4^ CT-2A-CXADR-BFP cells resulted in complete mortality by day 27 (median 25.5 days; Figure 4b), paralleling previously reported outcomes with parental CT-2A lines [32,33].

Previous studies on adenovirus infection have shown that CXADR is critical for viral attachment and internalization in multiple tumor types, including human gliomas and astrocytomas [47,48]. Consistently, transgenic models expressing human CXADR, either alone or together with other adenoviral receptors (CD46, DSG2), dramatically enhanced adenoviral entry and replication efficiency [49]. While prior work on B16-CXADR cells focused on their altered metastatic potential rather than viral responsiveness [50], our study extends this CXADR-humanization approach to Coxsackievirus infection, validating these humanized murine models as relevant tools for studying CVB-host interactions.

In this study, we compared local versus systemic delivery routes for LEV14. While intratumoral injection ensures high viral titers at the tumor site, its clinical application is constrained by the invasive nature of the procedure and the limited accessibility of deeply located gliomas. Conversely, intravenous administration enables noninvasive systemic delivery with the potential to target multifocal or disseminated lesions, though it overcomes several biological barriers, including rapid clearance, neutralizing antibodies, and limited extravasation from the vascular compartment [51]. Consistent with these known hurdles, our therapeutic experiments in the orthotopic glioma model were performed using intratumoral dosing. Nevertheless, IHC detection of LEV14 in orthotopic tumors after intravenous administration (Figure 6) provides proof-of-access and supports further evaluation of systemic LEV14 dosing as a potential complement or alternative to intratumoral delivery for glioma therapy.

One major challenge in systemic oncolytic virotherapy for gliomas is the blood–brain barrier (BBB), a highly selective interface that restricts transport between the vascular and central nervous system (CNS) compartments. The BBB severely limits penetration of therapeutic agents, including oncolytic viruses (OVs) [52]. In high-grade gliomas, partial BBB disruption can occur due to vascular invasion, hypoxia-induced angiogenesis, and VEGF activation [53,54,55]. Accordingly, most OVs in current clinical trials are delivered locally to bypass this barrier and achieve sufficient intratumoral concentrations [52]. Nevertheless, picornaviruses such as Coxsackieviruses could pass through the BBB. Their small capsid size (~30 nm) permits passage across even an intact BBB [56,57], and BBB disruption in tumor areas further enhances viral entry into the CNS.

The Coxsackieviruses studied in this paper exhibit strong selectivity towards tumors and an excellent safety profile. These viruses, derived from non-pathogenic enteroviral vaccine strains, replicate in malignant cells but not in normal tissues, thus effectively mitigating neurovirulence concerns that often limit systemic OV applications [13]. The safety of enteroviral vaccine strains has been well-documented during their usage for decades, stemming from their prior clinical use to prevent viral infections through interference with pathogenic viruses [12,13,58]. In our study, intravenous administration of LEV14 at high doses caused no mortality or notable signs of toxicity in mice. In previous work, histological examination confirmed the absence of pathological changes in major organs, including the brain, liver, spleen, and lungs, while the treated animals maintained normal weight gain [59]. The viral strains chosen for this work, therefore, combined both safety and high oncolytic potential. Coxsackieviruses B3, B5, and B6, belonging to *Enterovirus B* within the *Picornaviridae* family, are non-enveloped, single-stranded, (+)-RNA viruses that replicate exclusively in the cytoplasm. These viruses have been previously described and tested for their ability to selectively replicate in and destroy various tumor types, demonstrating promising antitumor activity [8,9,15,60]. The LEV14 strain (GenBank: MG642820.1) shares 82.47% nucleotide homology with the CVB5 Faulkner strain and 79.12% homology with the swine vesicular disease virus (SVDV) [59]. Moreover, its P1 region retains over 97% amino acid identity with CVB5 strains RO-14-5-70 (97.27%) and Faulkner (96.02%) [61]. Similarly, LEV15 strain (GenBank: JQ041368.1) exhibits nearly complete identity with other attenuated CVB6 strains, including 99.18% with Schmitt (AAF12719) [60].

Another limitation of intravenous virotherapy in murine models is the timing of tumor vascularization. At early stages of tumor development, the immature vasculature hampers virus access to all tumor cells, thus narrowing the therapeutic window. Effective treatment becomes possible only after sufficient vascularization, but initiation of later stages may fail to curb rapid glioma growth. In the CT-2A model, significant vascularization typically emerges by weeks 3–4 post-implantation [5,19], when tumors may already be too large for therapeutic benefit. However, many gliomas employ vessel co-option (VCO) rather than angiogenesis, hijacking pre-existing vasculature to sustain growth [62,63,64]. This process, observed in C6, CNS-1, GL261, and human GB models, provides early vascular functionality. The C6 model offered early evidence of VCO, revealing vascularized 1 mm tumors at one week post-implantation [62,64,65,66]. Similarly, GL261 gliomas showed extensive perivascular invasion within 96 h [67], with VCO detectable as early as one week [68,69]. Our study reinforces this concept, as IHC analysis confirmed that LEV14 successfully reached intracranial tumors via these existing vascular networks (Figure 6).

The formation of neutralizing antibodies represents another challenge for repeated systemic virotherapy, as circulating antibodies can reduce viral persistence [51]. In this study, neutralizing antibodies developed after the second LEV14 administration, regardless of delivery route (intravenous or intratumoral), suggesting comparable immunogenicity (Figure S12). Despite their appearance, therapeutic efficacy was preserved, as reflected in prolonged survival and smaller tumor volumes (Figure 4c,f,g).

The increase in tumor-infiltrating CD8+ T cells (Figures S13 and S18) and systemic cytokine changes (Figure S19) following LEV14 therapy suggests that tumor control in our models likely depends not only on direct viral oncolysis but also on adaptive T cell responses. Although T cell depletion experiments were beyond the scope of this study, they represent a logical next step to define the contributions of CD4+ and CD8+ subsets to LEV14 efficacy. Such depletion, while potentially reducing antiviral adaptive immunity and thus allowing greater viral persistence and oncolysis, might simultaneously abrogate long-term antitumor immunity, as demonstrated in oncolytic adenovirus models [70]. Conversely, oncolytic viruses can reshape PD-1/PD-L1 signaling by triggering proinflammatory cytokine release, which increases PD-L1 expression on immune cells and creates a rationale for combination with immune checkpoint inhibitors [71]. Indeed, multiple studies demonstrate that PD-1/PD-L1 blockade synergizes with virotherapy, enhancing antitumor immunity and improving therapeutic outcomes by relieving T cell exhaustion and amplifying virus-induced T cell priming [72,73,74]. However, careful scheduling of combination therapy is critical, as early PD-1 blockade may accelerate viral clearance and reduce intratumoral spread [75]. Rationally designed LEV14 combinations with temporally optimized PD-1 blockade, combined with mechanistic T cell depletion studies, therefore represent a promising strategy to further enhance therapeutic efficacy.

## 5. Conclusions

Thus, we established CXADR-humanized syngeneic tumor models as relevant platforms for evaluating CXADR-dependent human oncolytic enteroviruses in immunocompetent settings. These models retain key characteristics of the parental lines while exhibiting markedly increased sensitivity to group B Coxsackievirus strains, providing a more representative platform for preclinical glioblastoma research.

## Data Availability

The original contributions presented in this study are included in the article/Supplementary Materials. Further inquiries can be directed to the corresponding authors.

## Supplementary Materials

The following supporting information can be downloaded at: https://www.mdpi.com/article/10.3390/cancers18081280/s1, Figure S1: Schematic map of the lentiviral construct pLCMV-CXADR-C-tagBFP-puro; Table S1: The list of primers used for human *CXADR*, *RPL-19*, and murine *β-Actin* genes expression analysis by quantitative Real-Time PCR; Table S2: The list of primers used for *CXADR* gene identification by Sanger sequencing; Figure S2: ICC staining for CXADR expression in humanized murine cell lines; Figure S3: Representative morphological images of parental and humanized CT-2A, GL261, and B16 cell lines; Figure S4: CXADR expression in humanized cell lines; Video S1: Visualization of cytopathic effects in CT-2A, CT-2A-CXADR-BFP, and control HEK293T-ΔIFNAR1 cell lines; Video S2: Visualization of cytopathic effects in GL261, GL261-CXADR-BFP, and control HEK293T-ΔIFNAR1 cell lines; Figure S5: Increased susceptibility of humanized cell lines to CVBs infection; Figure S6: Survival curves of mice bearing intracranial CT-2A-CXADR-BFP, intracranial GL261-CXADR-BFP, and subcutaneous B16-CXADR-BFP tumors at indicated cell numbers; Figure S7: Histological analysis of CT-2A-CXADR-BFP tumors after subcutaneous implantation; Figure S8: Histological analysis of B16-CXADR-BFP tumors after subcutaneous implantation; Figure S9: Histological analysis of CT-2A, CT-2A-BFP, and CT-2A-CXADR-BFP tumors after intracranial implantation; Figure S10: T2-weighted MRI images of orthotopic glioma lesions; Figure S11: Histological analysis of GL261-CXADR-BFP tumors after intracranial implantation; Figure S12: Neutralizing activity of control and LEV14 antisera from mice bearing intracranial and subcutaneous CT-2A-CXADR-BFP tumors; Figure S13: Intracranial CT-2A-CXADR-BFP tumor immune infiltration post-LEV14 treatment; Figure S14: The results of histological examination of CT-2A-CXADR-BFP subcutaneous glioma after intravenous therapy with LEV14; Figure S15: Peripheral blood immune dynamics in mice bearing subcutaneous CT-2A-CXADR-BFP tumors during intravenous LEV14 therapy; Figure S16: Oncolytic activity of LEV14 in a subcutaneous B16-CXADR-BFP melanoma model; Figure S17: Histological examination of B16-CXADR-BFP melanoma after intravenous LEV14 therapy; Figure S18: B16-CXADR-BFP tumor immune infiltration post-LEV14 treatment; Figure S19: Cytokine analysis of serum samples from subcutaneous or intracranial CT-2A-CXADR-BFP tumor-bearing mice during four LEV14 administrations, compared to untreated controls.

## Abbreviations

The following abbreviations are used in this manuscript:

Ad5	Adenovirus serotype 5
BBB	Blood–brain barrier
CNS	Central nervous system
CPE	Cytopathic effect
CVB3	Coxsackievirus B3
CVB5	Coxsackievirus B5
CVB6	Coxsackievirus B6
CVBs	Group B Coxsackieviruses
CXADR	Coxsackievirus and Adenovirus Receptor
GB	Glioblastoma
H&E	Hematoxylin and eosin
ICC	Immunocytochemistry
IHC	Immunohistochemistry
i.t.	Intratumoral
i.v.	Intravenous
LEV	Live Enterovirus Vaccine
MOI	Multiplicity of infection
MRI	Magnetic resonance imaging
OVs	Oncolytic viruses
TCID50	50% tissue culture infectious dose
VCO	Vessel co-option
VEGF	Vascular endothelial growth factor
VSV	Vesicular stomatitis virus
