# Supplementary material for: Humanized Murine Glioblastoma Models for Evaluation of Coxsackievirus Oncolytic Therapy
Source: Cancers (Basel). 2026 Apr 17;18(8):1280. doi: 10.3390/cancers18081280 (PMC13114285; doi:10.3390/cancers18081280)
Supplement: Supplementary file 1 [file cancers-18-01280-s001.zip › cancers-4197290-supplementary.pdf]

Supplementary Materials

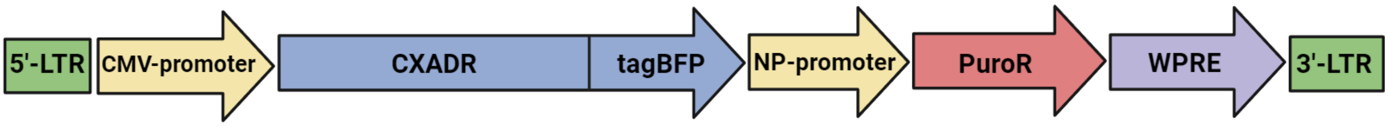

Figure S1. Schematic map of the lentiviral construct pLCMV-CXADR-C-tagBFP-puro.

Table S1. The list of primers used for human *CXADR*, *RPL-19*, and murine *β-Actin* genes expression analysis by quantitative Real-Time PCR.

Target	Orientation	Nucleotide sequence	Primer length
CXADR#1	Forward	5'-gcttgctctagcgctcattggt-3'	22
CXADR#1	Reverse	5'-gctctttggaggtggcacatct-3'	22
CXADR#2	Forward	5'-gtctgactcacagaaaatgcccac-3'	24
CXADR#2	Reverse	5'-ccagagtactcagaagaggcattttttaca-3'	30
mActb	Forward	5'-ctcctgagcgcaagtactctgtg-3'	23
mActb	Reverse	5'-taaaacgcagctcagtaacagtcc-3'	24
RPL19	Forward	5'-gagaccaatgaaatcgccaatg-3'	22
RPL19	Reverse	5'-gcggatgatcagcccatctt-3'	20

Table S2. The list of primers used for *CXADR* gene identification by Sanger sequencing.

Orientation	Nucleotide sequence	Primer length
Forward	5'-atggcgctcctgctgtgc-3'	18
Reverse	5'-tactatagacccatccttgctctgtgctg-3'	29

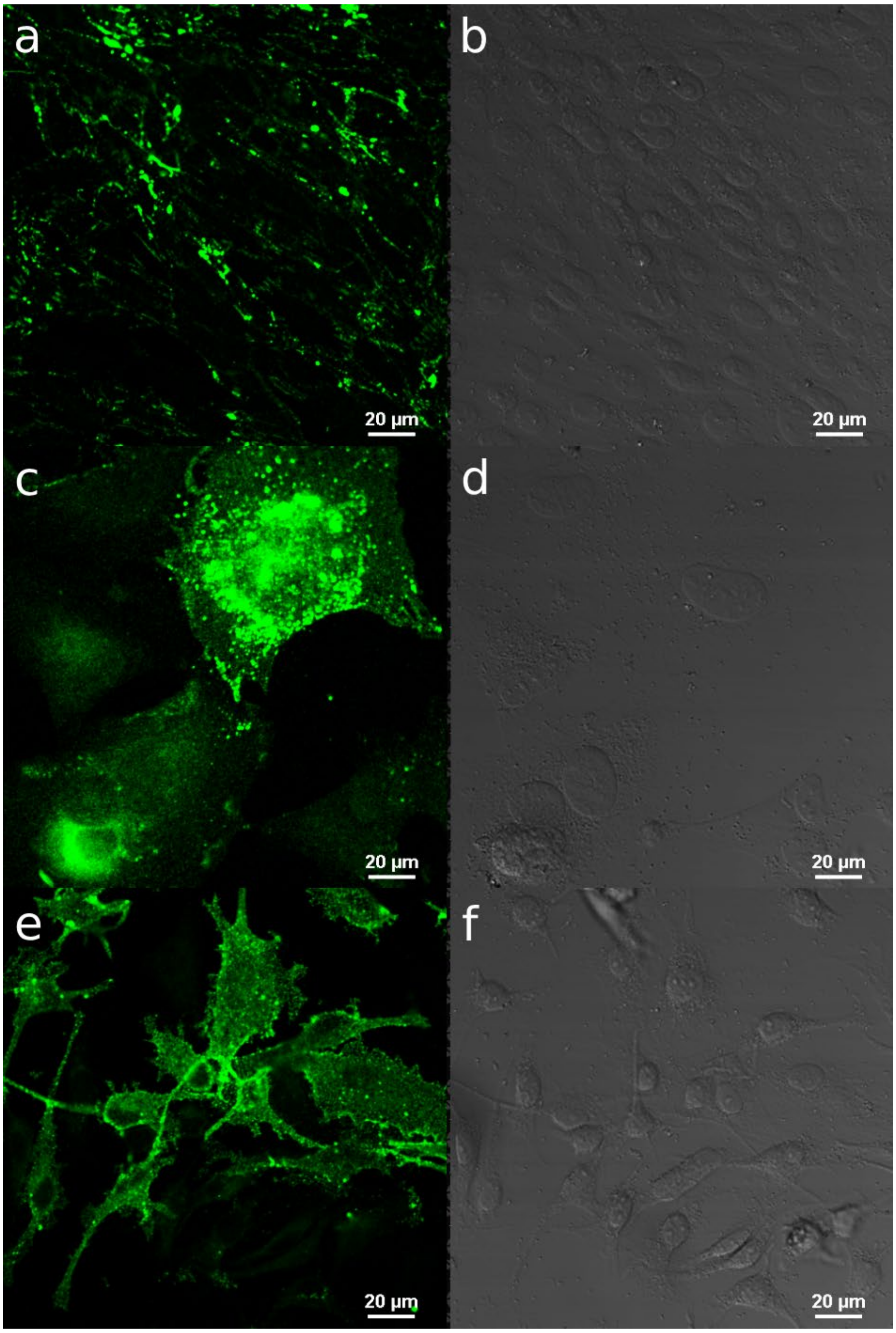

Figure S2. ICC staining for CXADR expression in humanized murine cell lines. (**a**,**b**) CT-2A-CXADR-BFP; (**c**,**d**) GL261-CXADR-BFP; (**e**,**f**) B16-CXADR-BFP.

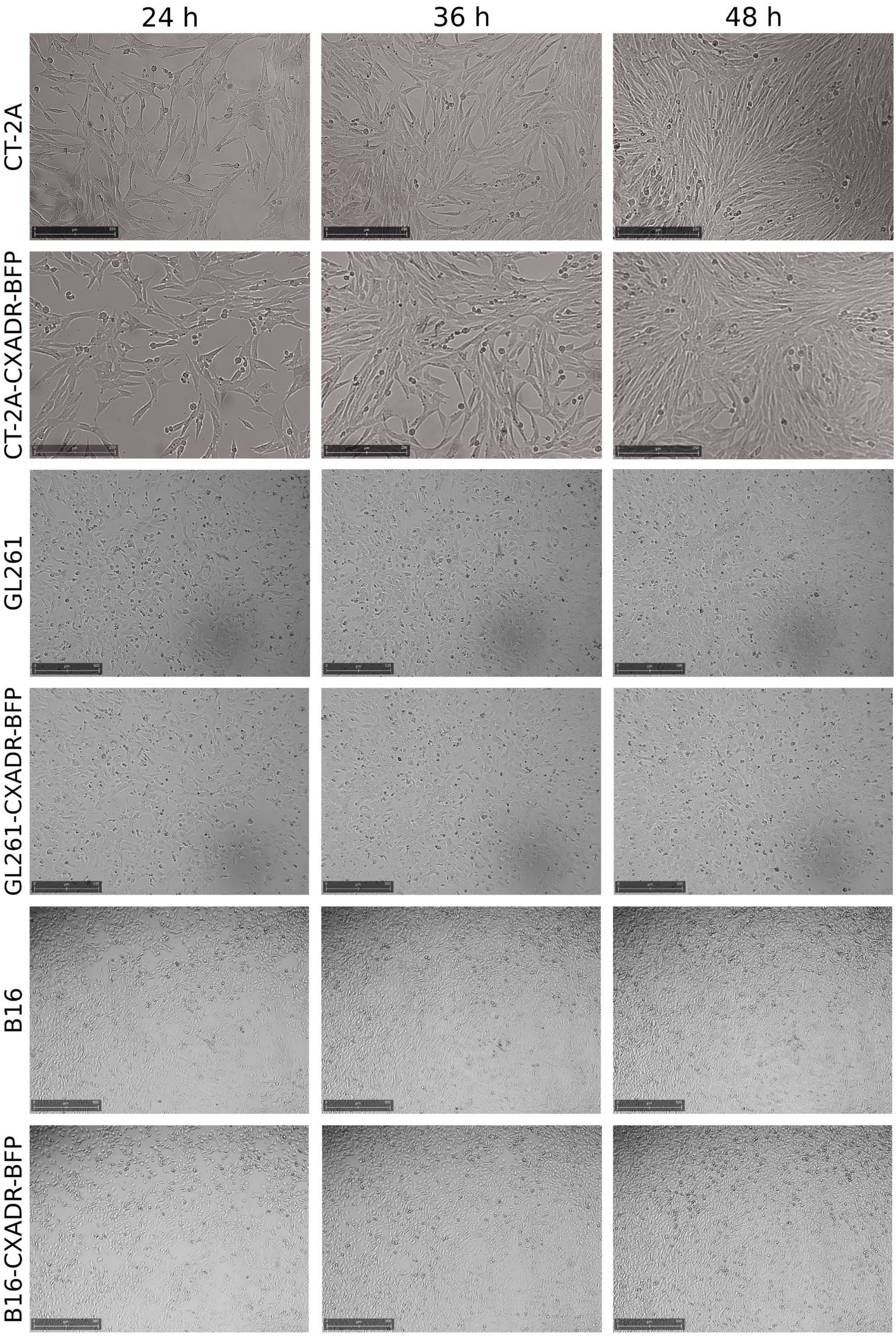

Figure S3. Representative morphological images of parental and humanized CT-2A, GL261, and B16 cell lines. Images were acquired 24, 36, and 48 h after seeding at equal confluence.

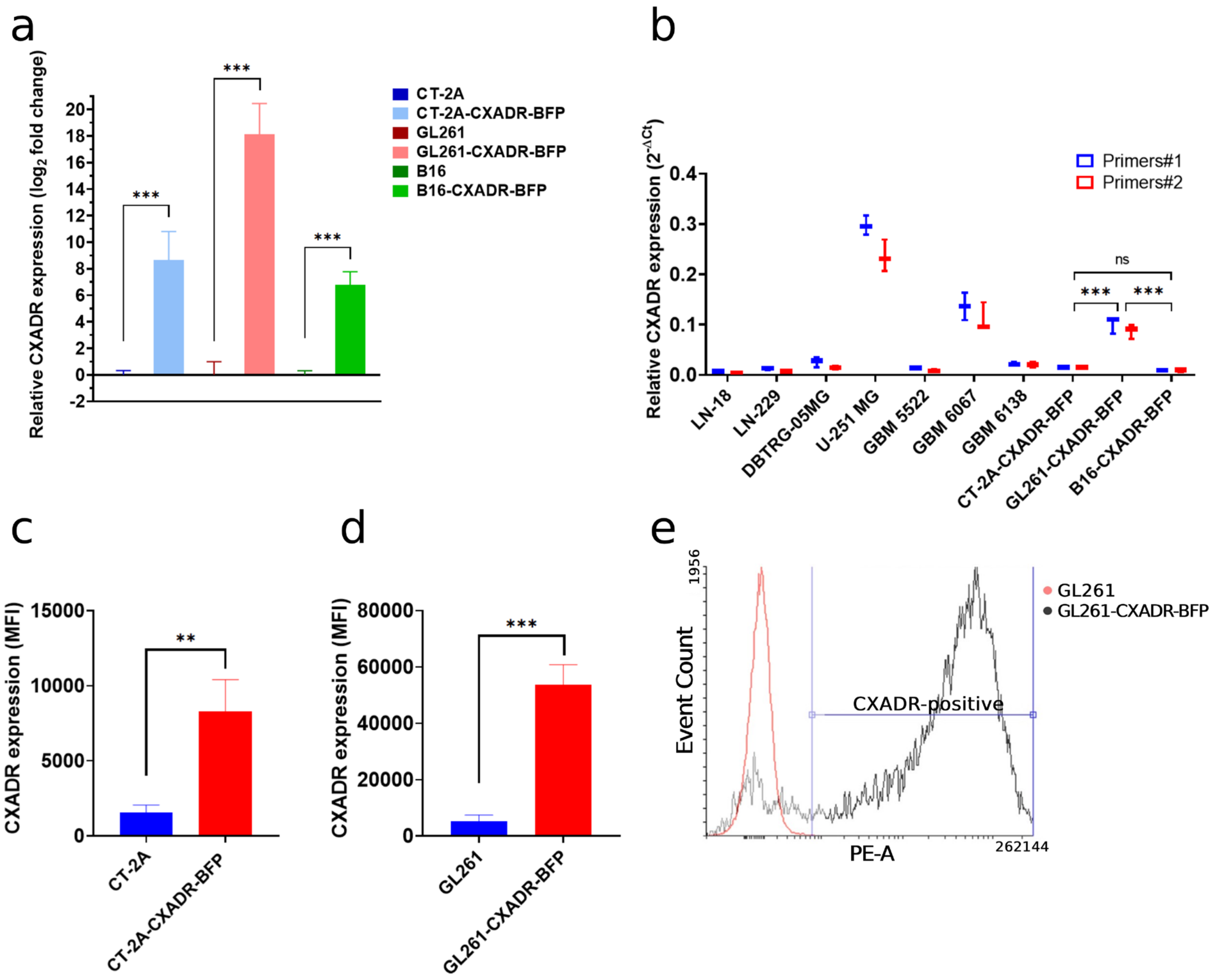

Figure S4. CXADR expression in humanized cell lines. (**a**) RT-qPCR quantification of human *CXADR* mRNA in parental and CXADR-transduced CT-2A, GL261, and B16 cell lines. Fold changes in gene expression were calculated using the $2^{-\Delta\Delta Ct}$ method. (**b**) Comparative RT-qPCR analysis of *CXADR* mRNA expression in model and primary human and humanized cell lines. (**c-e**) Flow cytometric analysis of surface CXADR expression represented as mean fluorescence intensity (MFI) in the PE channel. Values are means ± SD. Statistical analysis was performed using the Mann-Whitney U-test, ** $p < 0.01$, *** $p < 0.001$.

https://drive.google.com/file/d/1oAh_ypAmqXytx1O4ghcQJaP4I2wGgrNH/view?usp=sharing

Video S1. Visualization of cytopathic effects in CT-2A, CT-2A-CXADR-BFP, and control HEK293T-ΔIFNAR1 cell lines. Live-cell imaging within 48 h after infection with CVB3 (**a,e,i**), CVB5 (**b,f,j**), or VSV (**c,g,k**) at MOI 10. (**d,h,l**) – uninfected controls.

https://drive.google.com/file/d/12Q_eV43opQ3boOGIzPqZEV-yedSrdzgt/view?usp=sharing

Video S2. Visualization of cytopathic effects in GL261, GL261-CXADR-BFP, and control HEK293T-ΔIFNAR1 cell lines. Live-cell imaging within 72 h after infection with CVB5 (**a,d,g**) or VSV (**b,e,h**) at MOI 10. (**c,f,i**) – uninfected controls.

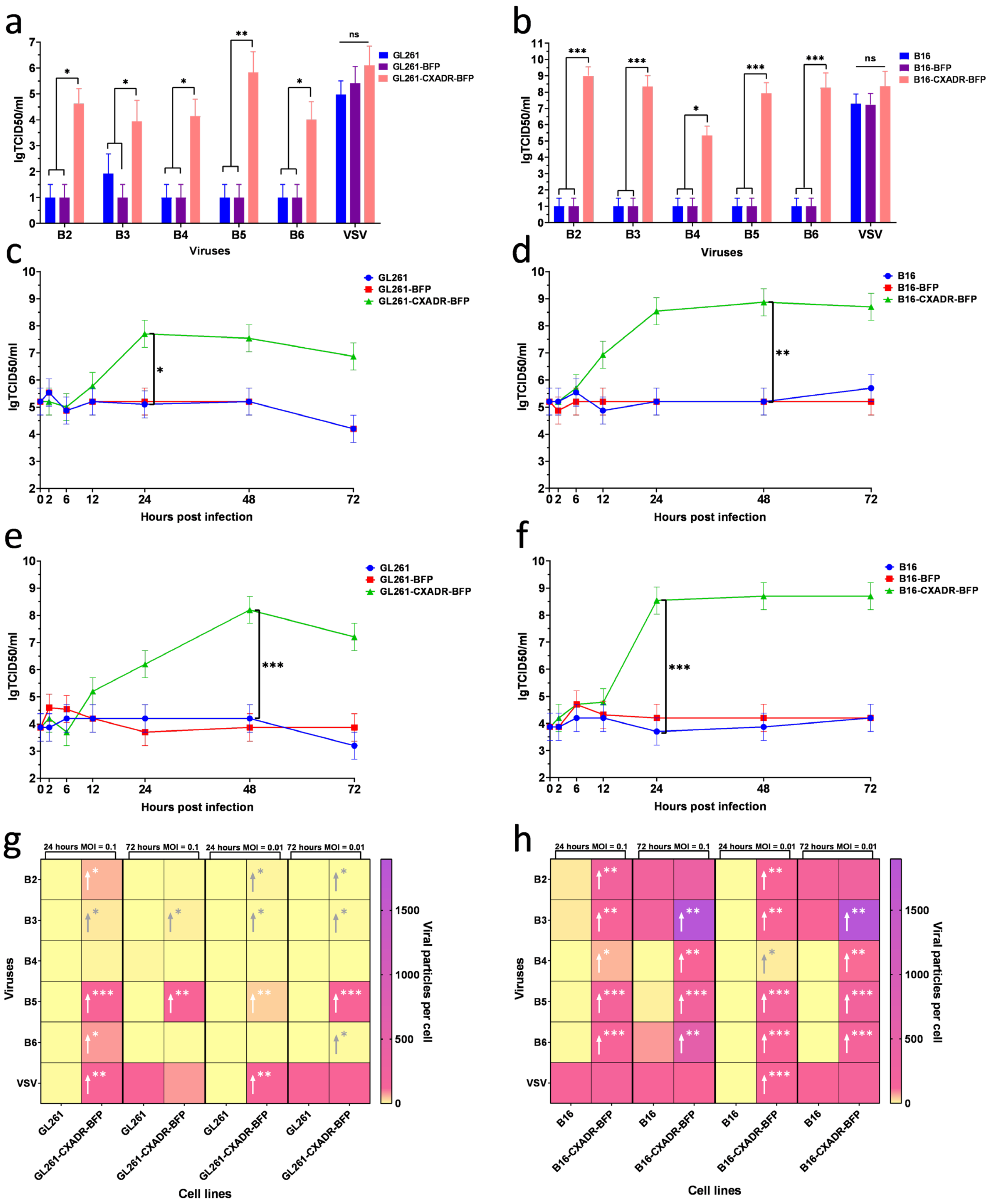

Figure S5. Increased susceptibility of humanized cell lines to CVBs infection. (**a-h**) Viral replication efficiency of CVBs and VSV in GL261, GL261-BFP, GL261-CXADR-BFP, B16, B16-BFP, and B16-CXADR-BFP cell lines. (**a**,**b**) Sensitivity of cells to viruses was evaluated at 72 h after infection. CVB5 replication was measured 2, 6, 12, 24, 48, and 72 h after infection at MOI 0.1 (**c**,**d**) or MOI 0.01 (**e**,**f**). (**g**,**h**) Viral replication of CVBs and VSV was measured at 24 h and 72 h after infection. The data are shown as means ± S.D of three independently related experiments (n = 3). Statistical analysis was performed using the Mann-Whitney U-test, * p<0.05, ** p<0.01, *** p<0.001.

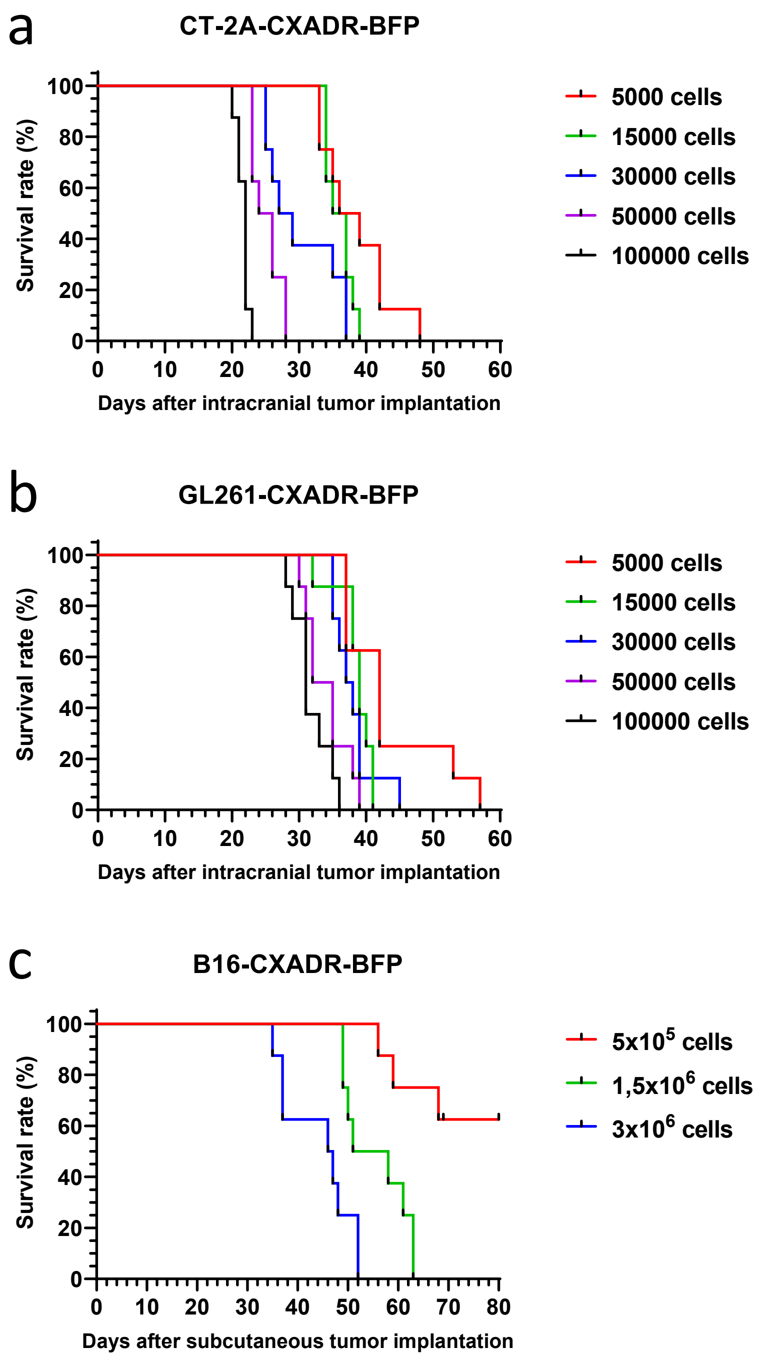

Figure S6. Survival curves of mice bearing intracranial CT-2A-CXADR-BFP (**a**), intracranial GL261-CXADR-BFP (**b**), and subcutaneous B16-CXADR-BFP (**c**) tumors at indicated cell numbers. Number of animals = 8 for each group.

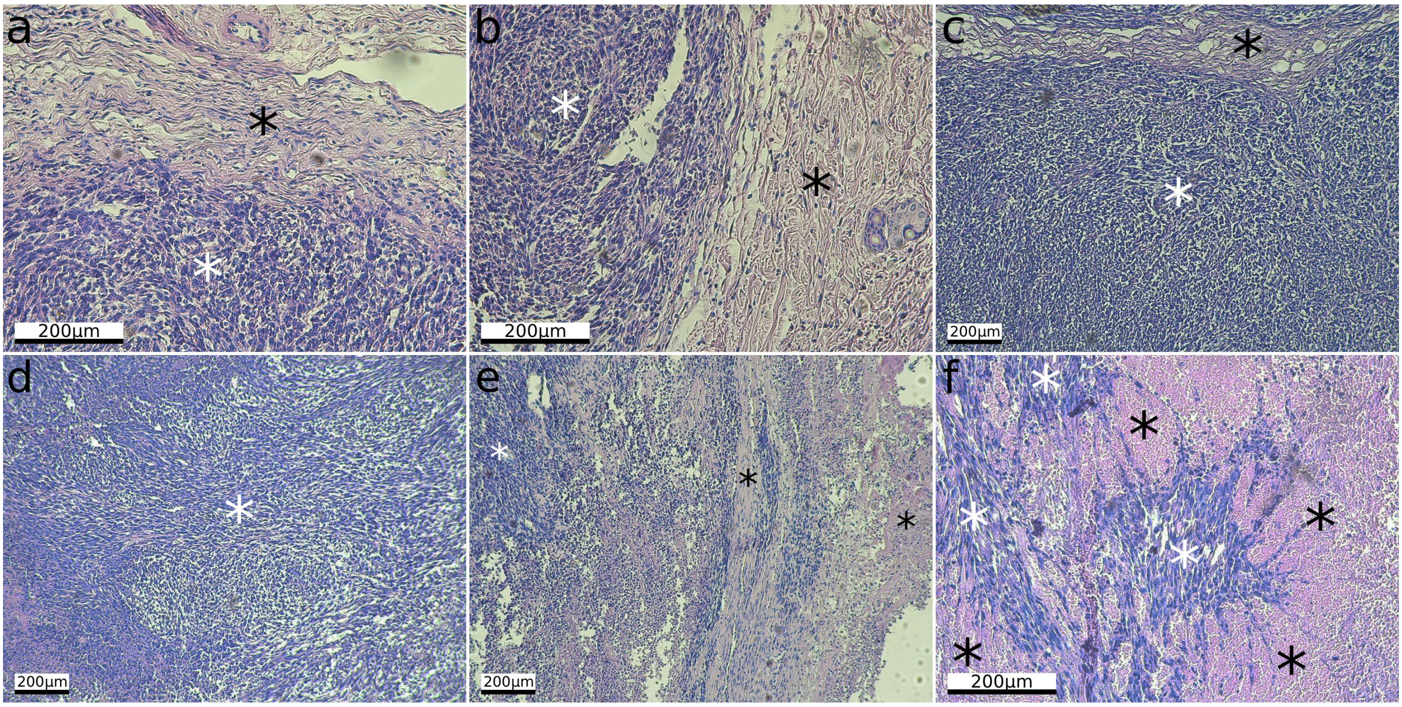

Figure S7. Histological analysis of CT-2A-CXADR-BFP tumors after subcutaneous implantation (H&E staining). Early time points (**a**,**b**, x20) and late time points (**c**-**e**, x10; **f**, x20). Early stages were analyzed up to 30 days post-implantation, whereas late stages corresponded to the endpoint of the observation period (up to 60 days post-implantation), when euthanasia was performed because tumors reached a critical volume. White asterisks denote tumor tissue; black asterisks indicate normal tissue adjacent to the tumor.

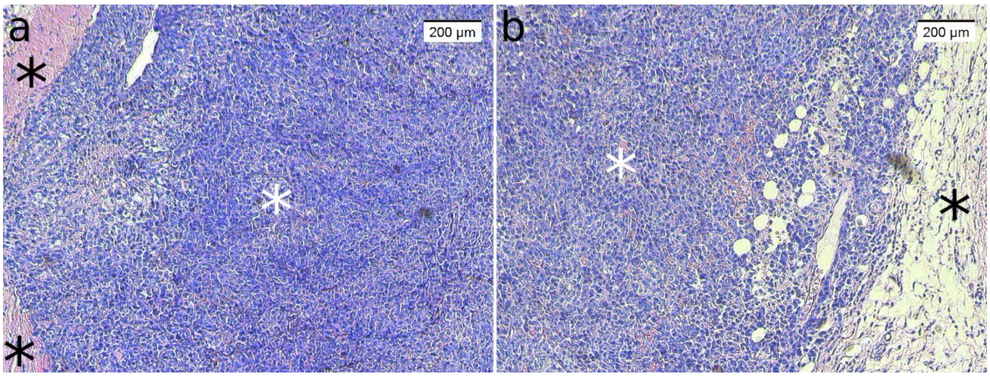

Figure S8. Histological analysis of B16-CXADR-BFP tumors after subcutaneous implantation (H&E staining, **a**,**b**, x10). White asterisks denote tumor tissue; black asterisks indicate normal tissue adjacent to the tumor.

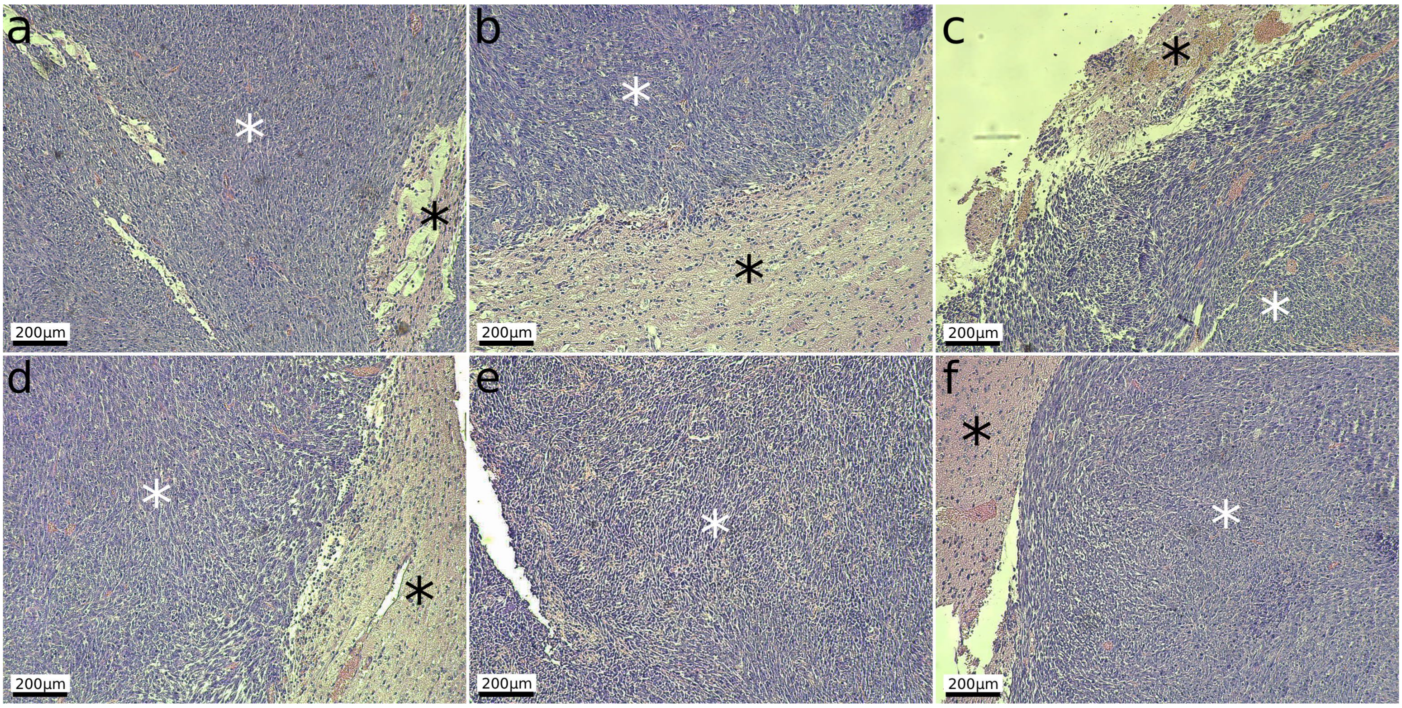

Figure S9. Histological analysis of CT-2A, CT-2A-BFP, and CT-2A-CXADR-BFP tumors after intracranial implantation (H&E staining, x10). (**a**,**b**) CT-2A; (**c**,**d**) CT-2A-BFP; (**e**,**f**) CT-2A-CXADR-BFP. White asterisks denote tumor brain tissue; black asterisks indicate normal brain tissue adjacent to the tumor.

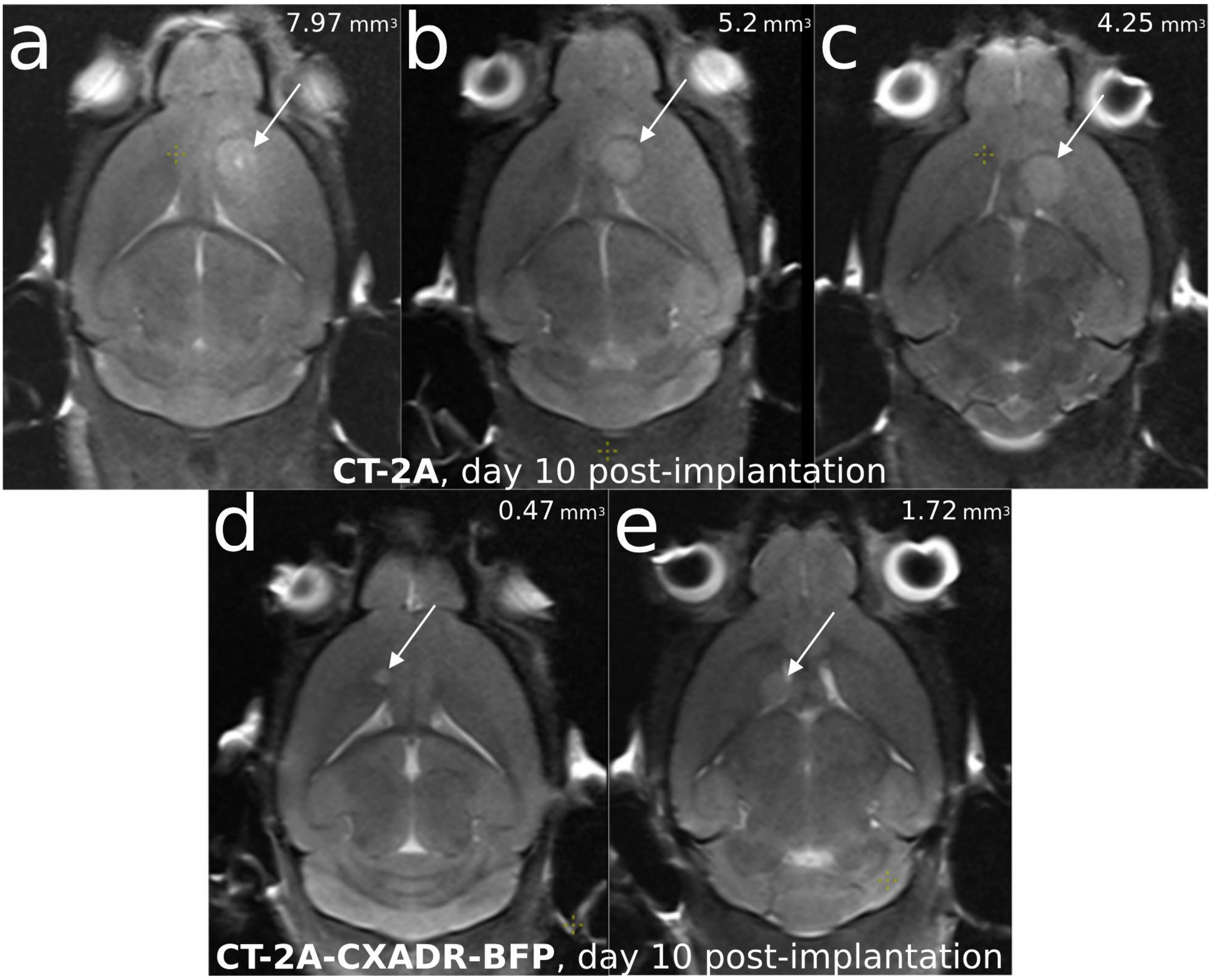

Figure S10. T2-weighted MRI images of orthotopic glioma lesions. Coronal MRI images of brains of mice with orthotopic tumors at 10th day post-implantation: (**a-c**) CT-2A, right hemisphere; (**d,e**) CT-2A-CXADR-BFP, left hemisphere. Tumor volumes were quantified from serial T2-weighted slices and are indicated in the upper right corner of each panel.

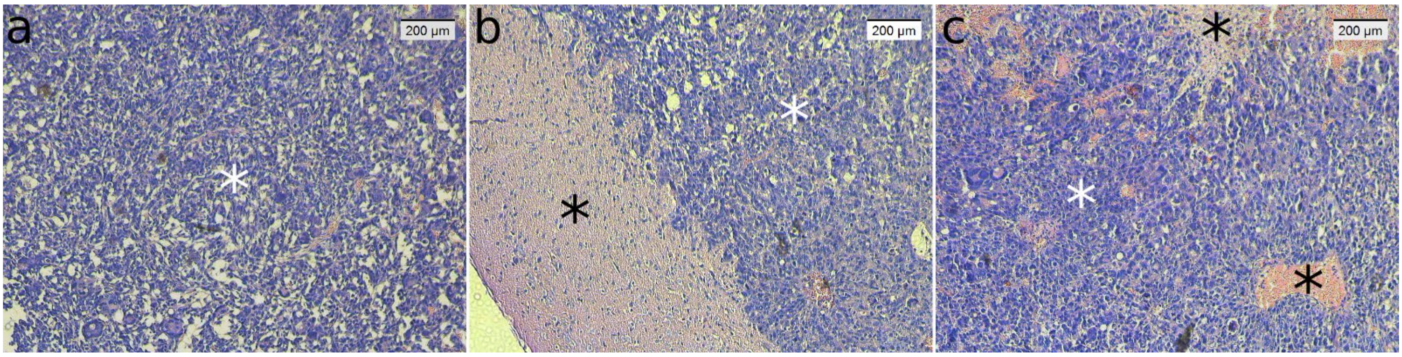

Figure S11. Histological analysis of GL261-CXADR-BFP tumors after intracranial implantation (H&E staining, **a-c**, x10). White asterisks denote tumor tissue; black asterisks indicate normal tissue adjacent to the tumor.

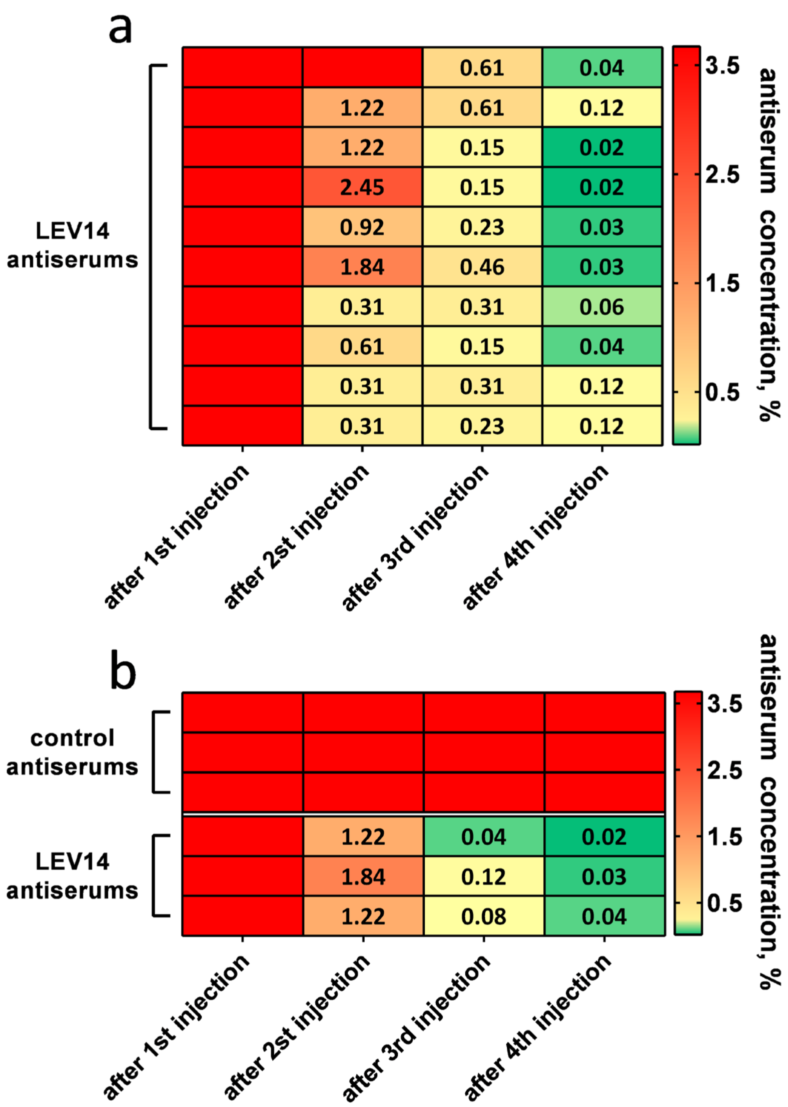

Figure S12. Neutralizing activity (minimum antiserum concentration at which more than 50% of cells remained viable) of control and LEV14 antisera from mice bearing intracranial (**a**) and subcutaneous (**b**) CT-2A-CXADR-BFP tumors. Cell viability was assessed 24 hours post-infection of HEK293T-ΔIFNAR1 cells with LEV14 (MOI 0.2) pre-incubated with antisera. Blood samples were collected from control and LEV14-treated mice 2 days after each virus injection. Each row in the table represents a distinct biological replicate.

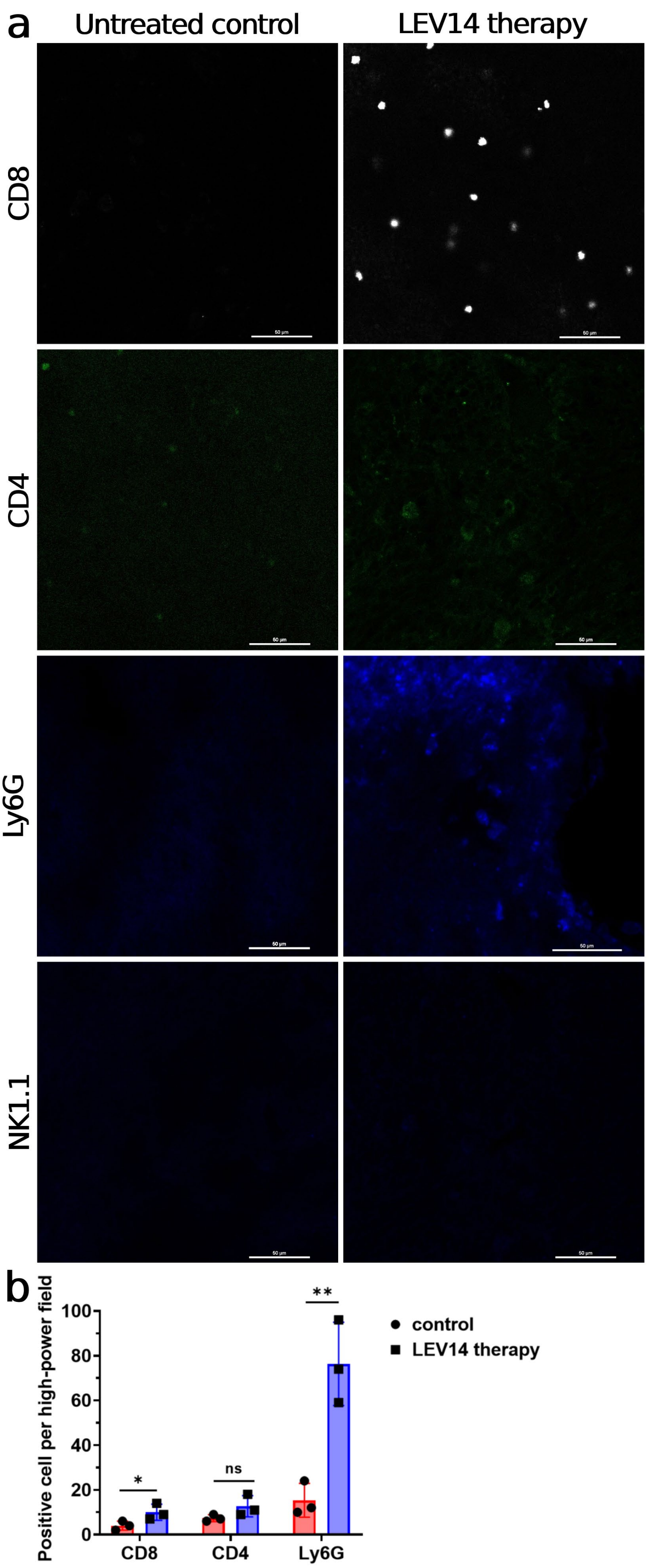

Figure S13. Intracranial CT-2A-CXADR-BFP tumor immune infiltration post-LEV14 treatment. (**a**) Representative tumor IHC sections stained for CD8, CD4, Ly6G, and NK1.1 4 days after the 4th intratumoral LEV14 injection. Scale bars, 50 μm. (**b**) Quantification of positive cells per high-power field, presented as mean ± SD. Statistical analysis was performed using the Mann-Whitney U-test, * $p<0.05$, ** $p<0.01$.

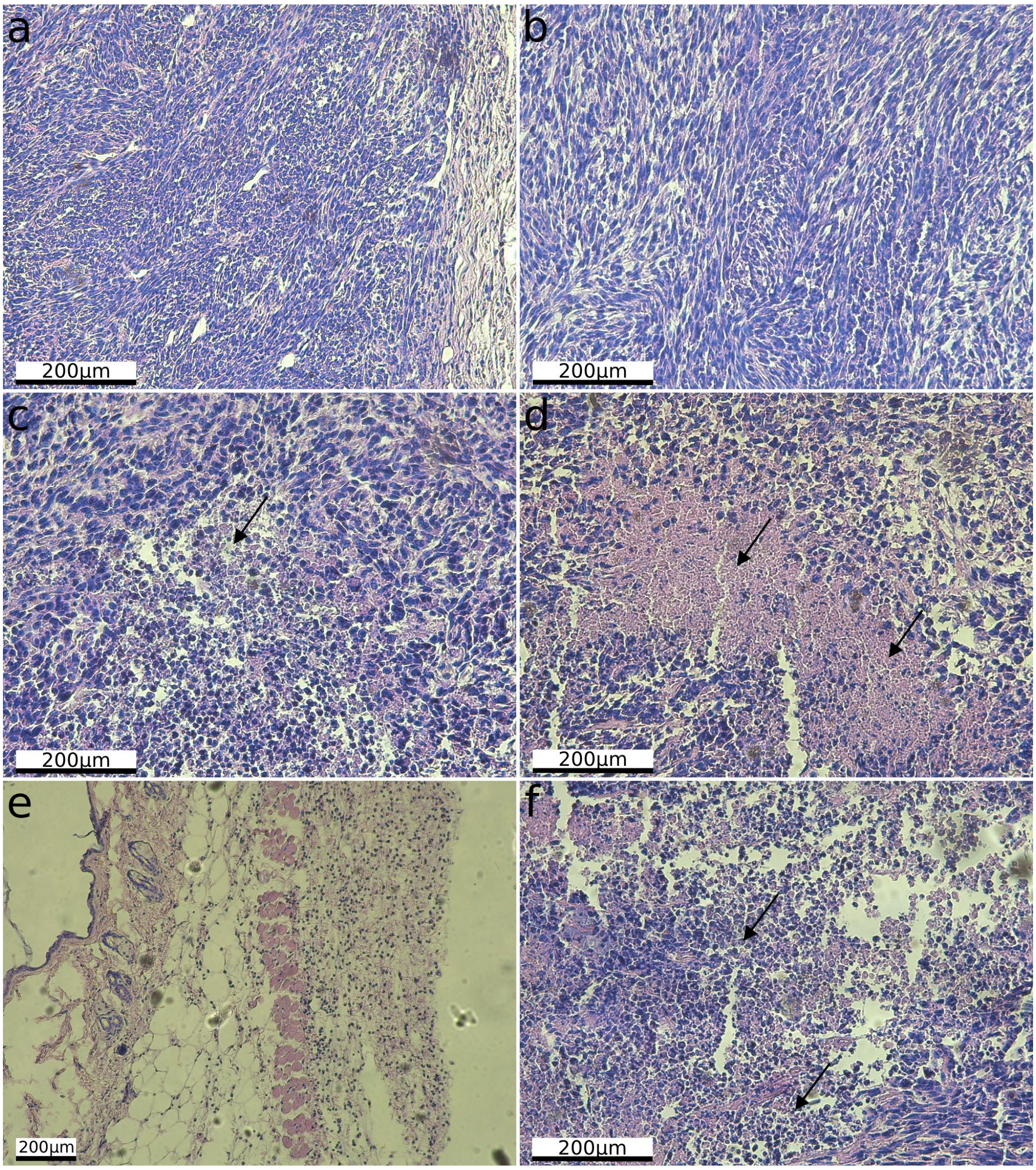

Figure S14. The results of histological examination (H&E staining) of CT-2A-CXADR-BFP subcutaneous glioma after intravenous therapy with LEV14. (**a**,**b**, x20) Brain sections from control mice without therapy. (**c**,**d**,**f**, x20; **e**, x10) Brain sections from mice treated with LEV14. Arrows indicate the most extensive areas of necrosis and regions with loss of cellular architecture.

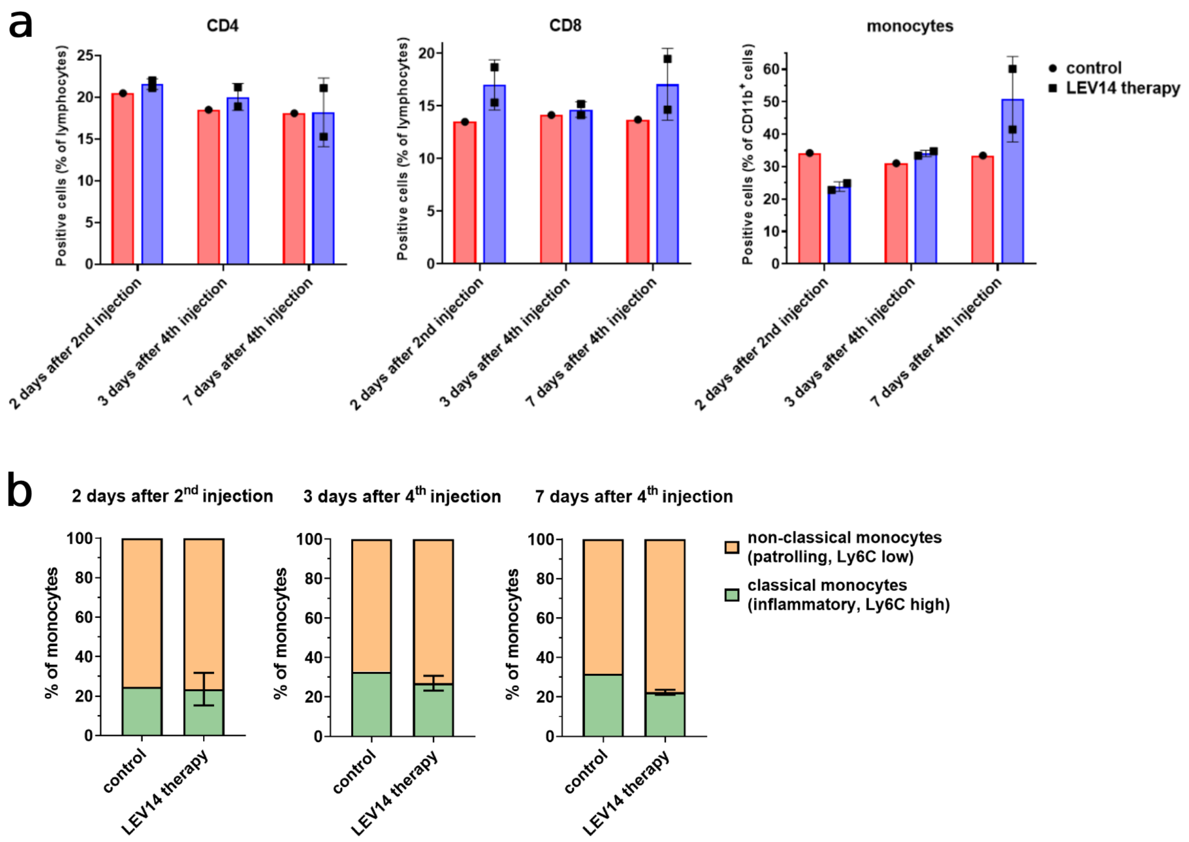

Figure S15. Peripheral blood immune dynamics in mice bearing subcutaneous CT-2A-CXADR-BFP tumors during intravenous LEV14 therapy. (**a**) Percentages of CD4+, CD8+ T cells, and total monocytes measured after the 2nd and the 4th LEV14 administrations. (**b**) Proportions of patrolling (Ly6C low) vs inflammatory monocyte (Ly6C high). Values are means ± SD.

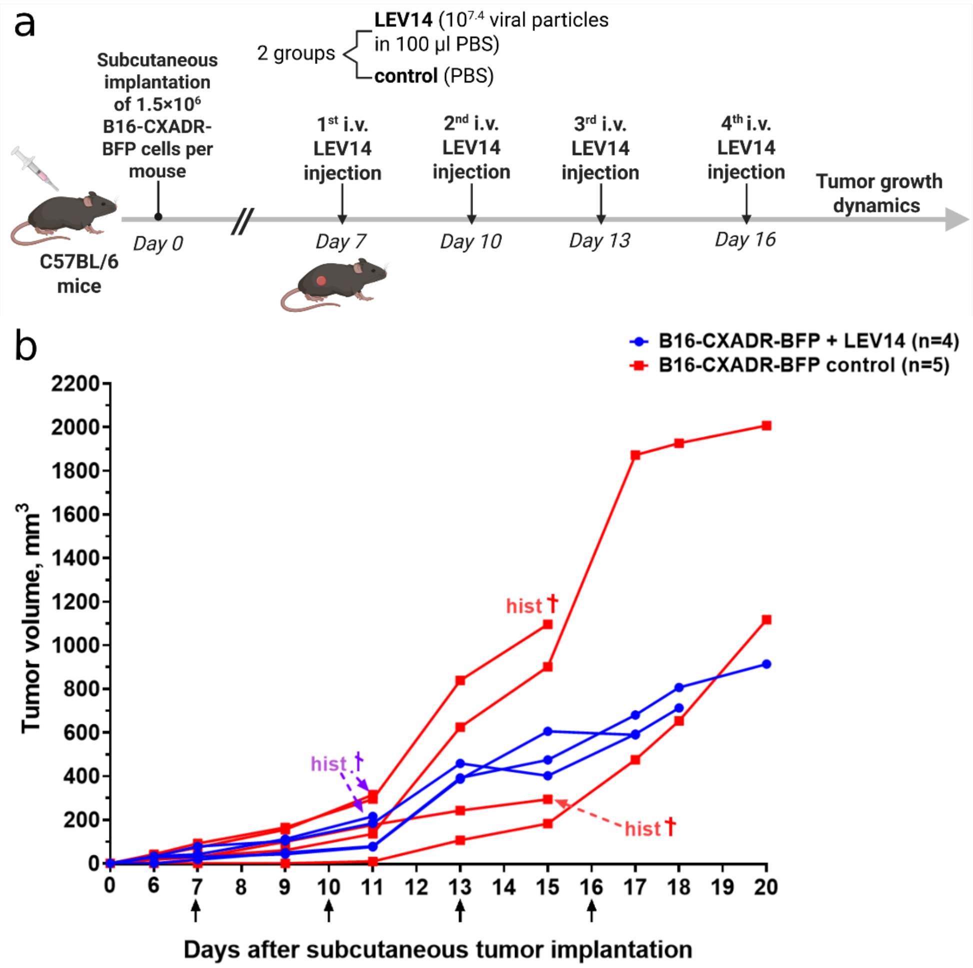

Figure S16. Oncolytic activity of LEV14 in a subcutaneous B16-CXADR-BFP melanoma model. (**a**) Treatment protocol scheme (created with Biorender.com). (**b**) Growth dynamics of B16-CXADR-BFP subcutaneous xenografts following four consecutive intravenous administrations of LEV14 (on days 7, 10, 13, and 16 post-implantation, $10^{7.4}$ TCID50 per mouse). "hist" indicates mice euthanized at the indicated time points for histological analysis. n, number of animals. Values are means ± SD. Statistical analysis was performed using the Mann-Whitney U-test.

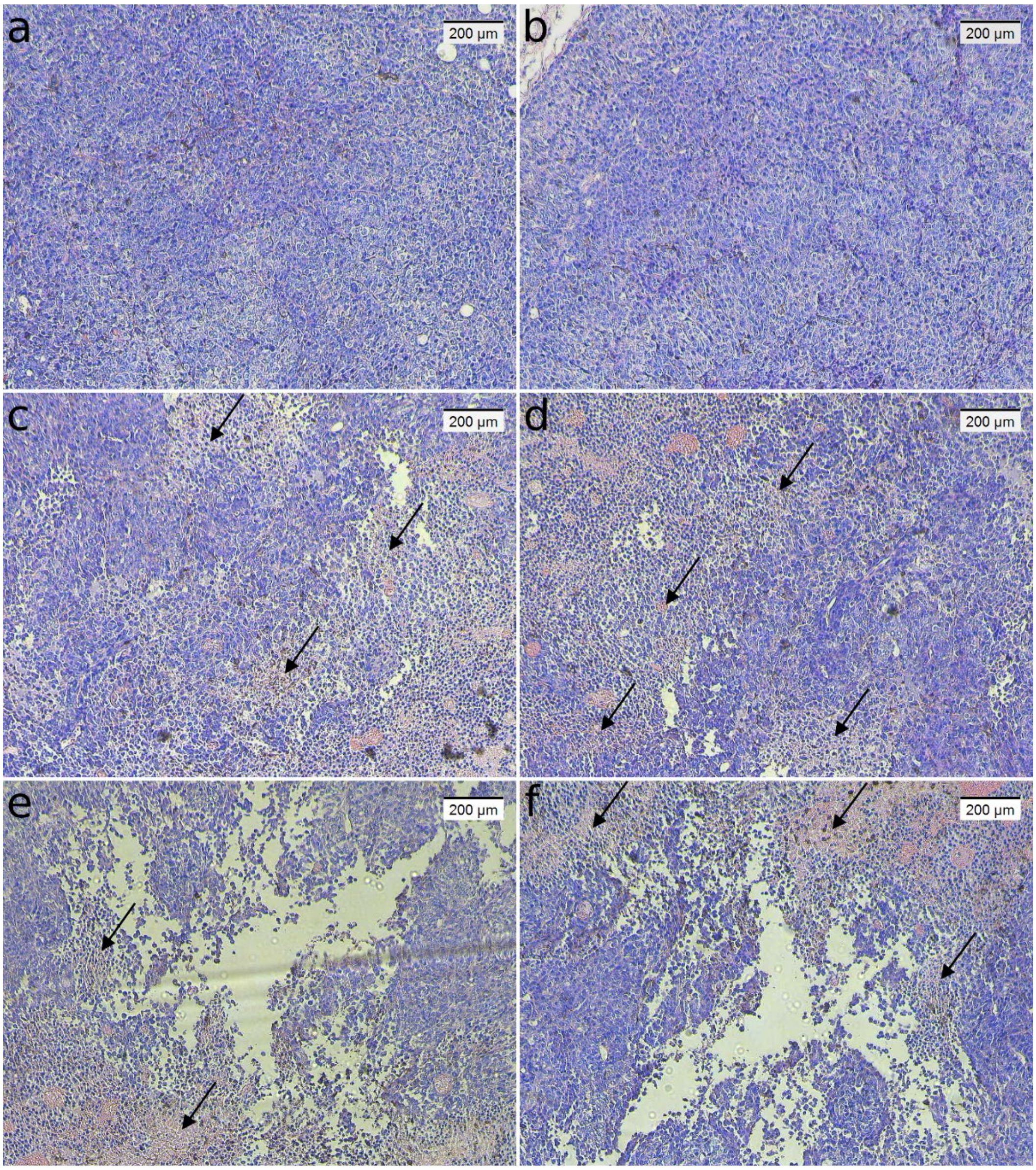

Figure S17. Histological examination (H&E staining) of B16-CXADR-BFP melanoma after intravenous LEV14 therapy (x10). (**a**,**b**) Control mice without therapy. (**c**-**f**) Mice treated with LEV14. Arrows indicate the most extensive areas of necrosis and regions with loss of cellular architecture.

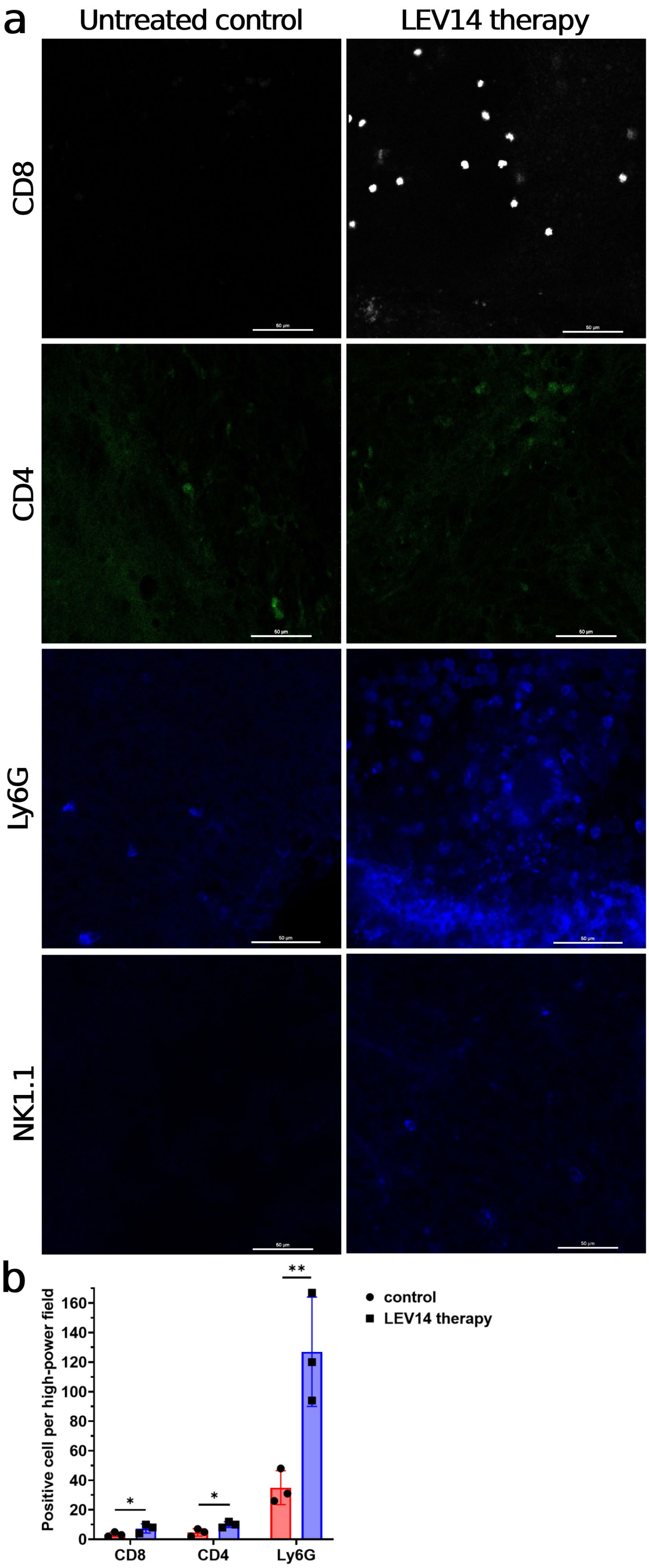

Figure S18. B16-CXADR-BFP tumor immune infiltration post-LEV14 treatment. (**a**) Representative tumor IHC sections stained for CD8, CD4, Ly6G, and NK1.14 days after the 4th intravenous LEV14 injection. Scale bars, 50 µm. (**b**) Quantification of positive cells per high-power field, presented as mean ± SD. Statistical analysis was performed using the Mann-Whitney U-test, * $p<0.05$, ** $p<0.01$.

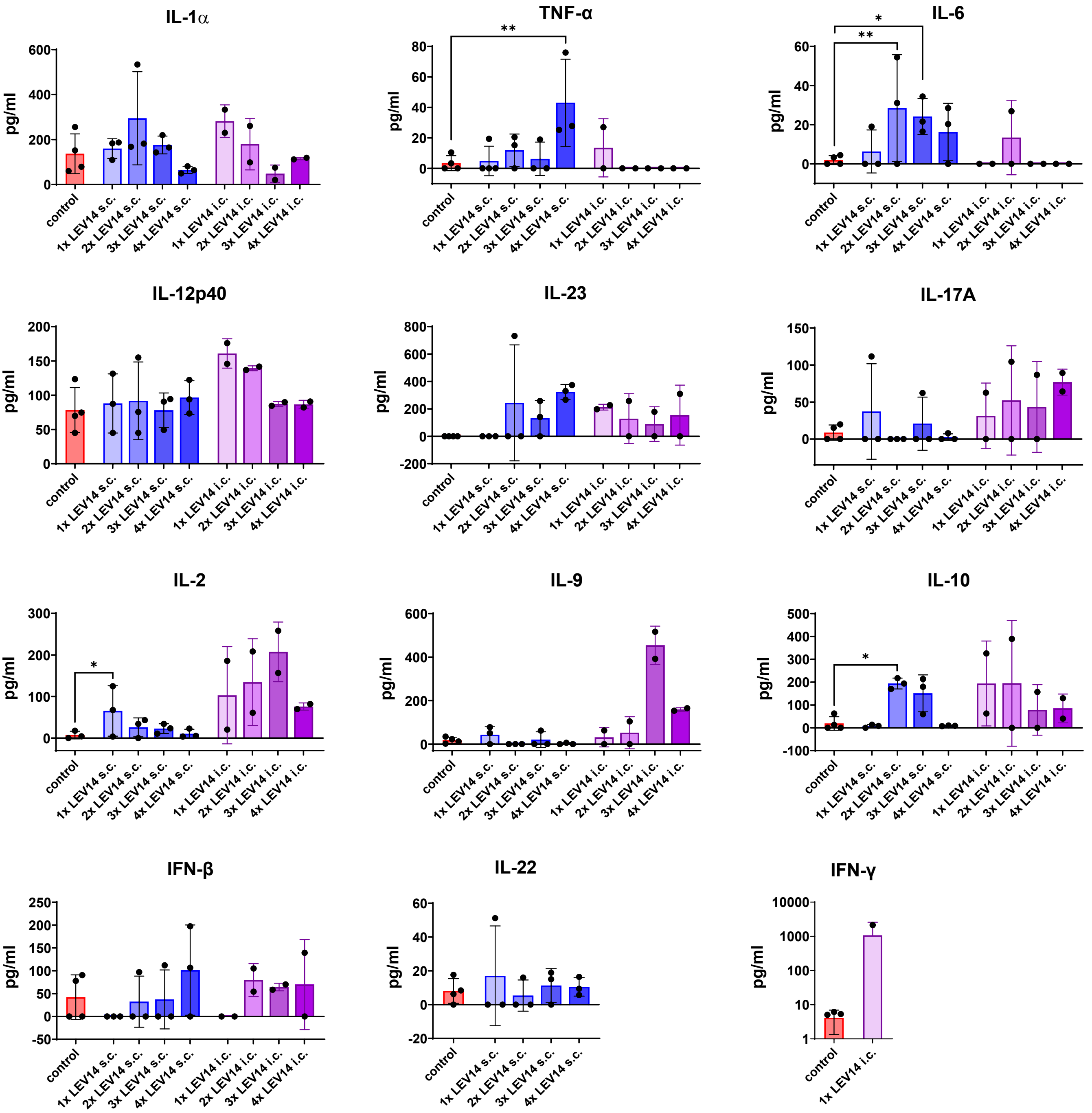

Figure S19. Cytokine analysis of serum samples from subcutaneous (s.c.) or intracranial (i.c.) CT-2A-CXADR-BFP tumor-bearing mice during four LEV14 administrations (intravenous for s.c. tumors, intratumoral for i.c. tumors), compared to untreated controls. Data shown as mean ± SD. Statistical analysis was performed using the Mann-Whitney U-test, * $p<0.05$, ** $p<0.01$.
